# CAT-Posterior Mean Site Frequencies Improves Phylogenetic Modeling Under Maximum Likelihood and Resolves Tardigrada as the Sister of Arthropoda Plus Onychophora

**DOI:** 10.1093/gbe/evae273

**Published:** 2024-12-23

**Authors:** Mattia Giacomelli, Matteo Vecchi, Roberto Guidetti, Lorena Rebecchi, Philip C J Donoghue, Jesus Lozano-Fernandez, Davide Pisani

**Affiliations:** Bristol Palaeobiology Group, School of Biological Sciences, Life Sciences Building, University of Bristol, Bristol, UK; Department of Genetics, Microbiology and Statistics, University of Barcelona, Barcelona, Spain; Institute of Systematics and Evolution of Animals, Polish Academy of Sciences, Krakow, Poland; Department of Life Sciences, University of Modena and Reggio Emilia, Modena, Italy; Department of Life Sciences, University of Modena and Reggio Emilia, Modena, Italy; Bristol Palaeobiology Group, School of Earth Sciences, Life Sciences Building, University of Bristol, Bristol, UK; Department of Genetics, Microbiology and Statistics, University of Barcelona, Barcelona, Spain; Bristol Palaeobiology Group, School of Biological Sciences, Life Sciences Building, University of Bristol, Bristol, UK; Bristol Palaeobiology Group, School of Earth Sciences, Life Sciences Building, University of Bristol, Bristol, UK

**Keywords:** phylogenomics, Tardigrada, Ecdysozoa, model adequacy tests, parametric bootstrap

## Abstract

Tardigrada, the water bears, are microscopic animals with walking appendages that are members of Ecdysozoa, the clade of molting animals that also includes Nematoda (round worms), Nematomorpha (horsehair worms), Priapulida (penis worms), Kinorhyncha (mud dragons), Loricifera (loricated animals), Arthropoda (insects, spiders, centipedes, crustaceans, and their allies), and Onychophora (velvet worms). The phylogenetic relationships within Ecdysozoa are still unclear, with analyses of molecular and morphological data yielding incongruent results. Accounting for across-site compositional heterogeneity using mixture models that partition sites in frequency categories, CATegories (CAT)-based models, has been shown to improve fit in Bayesian analyses. However, CAT-based models such as CAT-Poisson or CAT-GTR (where CAT is combined with a General Time Reversible matrix to account for replacement rate heterogeneity) have proven difficult to implement in maximum likelihood. Here, we use CAT-posterior mean site frequencies (CAT-PMSF), a new method to export dataset-specific mixture models (CAT-Poisson and CAT-GTR) parameterized using Bayesian methods to maximum likelihood software. We developed new maximum likelihood-based model adequacy tests using parametric bootstrap and show that CAT-PMSF describes across-site compositional heterogeneity better than other across-site compositionally heterogeneous models currently implemented in maximum likelihood software. CAT-PMSF suggests that tardigrades are members of Panarthropoda, a lineage also including Arthropoda and Onychophora. Within Panarthropoda, our results favor Tardigrada as sister to Onychophora plus Arthropoda (the Lobopodia hypothesis). Our results illustrate the power of CAT-PMSF to model across-site compositionally heterogeneous datasets in the maximum likelihood framework and clarify the relationships between the Tardigrada and the Ecdysozoa.

**Significance**

Tardigrades (water bears) are a phylum of invertebrate animals the relationships of which have proven hard to resolve. Here, we use a new tardigrade dataset to test the CAT-posterior mean site frequencies (CAT-PMSF) approach, a novel way to implement parameter-rich across-site compositional heterogeneous models in a likelihood framework. We develop a new parametric bootstrap-based pipeline to test the fit of models in a likelihood framework and show that CAT-PMSF describes the data better than other considered models, demonstrating that CAT-PMSF improves the modeling of across-site compositional heterogeneity in maximum likelihood and has the power to clarify difficult phylogenetic problems.

## Introduction

Uncovering the phylogenetic relationships of animals has proven difficult, and molecular phylogenetics has in some cases corroborated (Regier et al. 2005; Ontano et al. 2021) and in other cases challenged (Halanych et al. 1995; Aguinaldo et al. 1997; Delsuc et al. 2006) long-standing hypotheses of metazoan evolution. The discovery of Ecdysozoa (Aguinaldo et al. 1997), where analyses of molecular data united eight invertebrate phyla of little morphological similarity (arthropods, onychophorans, tardigrades, nematodes, nematomorphs, loriciferans, kinorhynchs, and priapulids), represented an inflection point in modern phylogenetics. The monophyly of Ecdysozoa has been widely corroborated in subsequent decades (Giribet and Edgecombe 2017). However, their internal relationships have proven difficult to resolve due to heterogeneous rates of molecular evolution within the group (Giribet and Edgecombe 2017) and a chronic imbalance in taxon sampling that has focused on economically and biomedically relevant lineages such as Arthropoda and Nematoda.

Based on morphology, a diversity of ecdysozoan phylogenies have been proposed, with three groups usually considered to represent monophyletic lineages (Nielsen 2012; Giribet and Edgecombe 2017). These are Scalidophora (Priapulida, Kinorynchya, and Loricifera), Nematoida (Nematoda plus Nematomorpha), and Panarthropoda (Tardigrada, Onychophora, and Arthropoda). However, the monophyly of these three groups and their interrelationships are far from certain. Uncertainty centers around the phylum Tardigrada which is consistently found as a member of Panarthropoda by morphology (e.g. Budd 2001; Edgecombe 2010; Legg et al. 2013; Yang et al. 2015; Kihm et al. 2023), because of their segmented body plan, paired walking appendages, alpha chitin in the cuticle, and ventral nerve cords. However, Tardigrada is unstable in phylogenomic analyses, where it emerges either a member of Panarthropoda or Nematoida (Dunn et al. 2008; Hejnol et al. 2009; Campbell et al. 2011; Borner et al. 2014; Laumer et al. 2019; Yoshida et al. 2017; Howard et al. 2022). Morphological apomorphies for tardigrades as a member of Nematoida are missing, but the loss of specific HOX genes has been proposed as a tentative molecular apomorphy for this group (Yoshida et al. 2017).

Molecular studies that have recovered Tardigrada as a member of Panarthropoda resolve tardigrades as the sister lineage of either Onychophora (“Protoarthropoda” hypothesis—Wägele et al. 1999; Rota-Stabelli et al. 2011) or more often, Onychophora plus Arthropoda (“Lobopodia” hypothesis; Campbell et al. 2011; Laumer et al. 2019; Howard et al. 2022). Morphological and gene expression evidence supporting Tardigrada as a member of Panarthropoda is commonplace, but either as sister to Arthropoda (“Tactopoda” hypothesis’; e.g. Nielsen et al. 1996; Budd 2001; Mayer et al 2013; Smith and Ortega-Hernández 2014; Yang et al. 2015, 2016; Howard et al. 2020; Lerosey-Aubril and Ortega-Hernández 2022) or as sister to Arthropoda plus Onychophora (“Lobopodia” hypothesis; e.g. Legg et al. 2012, 2013; Caron and Aria 2017, 2020; Zeng et al. 2020; Aria et al. 2021). Wu et al. (2023) showed that morphological datasets do not always have the power to statistically distinguish between Tactopoda and Lobopodia but, when they do, they tend to favor Lobopodia.

In an attempt to discriminate among these competing hypotheses, we assembled a phylogenomic dataset using the Benchmarked Universal Single-Copy Orthologs (BUSCO) metazoan dataset (Simão et al. 2015) and exploited recent advances in the implementation of across-site compositional heterogeneous models in a likelihood framework—the CAT-posterior mean site frequencies (CAT-PMSF) approach (Szánthó et al. 2023). CAT-PMSF allows the implementation of dataset-specific infinite mixture models, the Categories (CAT)-based models of Lartillot and Philippe (2004), to accommodate across-site compositional heterogeneity in a maximum likelihood framework. Other models exist to describe across-site compositional heterogeneity in maximum likelihood: the empirical mixture models with a fixed number of categories (C10 to C60) of Si Quang et al. (2008). However, infinite mixture models such as CAT-Poisson and CAT-GTR, which also uses a General Time Reversible matrix to accomodate replacement rate heterogeneity (e.g. Tihelka et al. 2021), have been shown to achieve greater fit using simulated and real data (Giacomelli et al. 2022). Unfortunately, for large datasests CAT-Poisson and CAT-GTR can be computationally too demanding (Wang et al. 2018; Szánthó et al. 2023). CAT-PMSF is a modification of the PMSF procedure developed by Wang et al. (2018) to approximate empirical mixture models with a fixed number of categories (C10 to C60) and reduce runtime and memory usage when performing analyses using models such as LG-CXX or GTR-CXX (where XX can be 10, 20, …, 60). The expected advantage of CAT-PMSF over CXX models, and over the original PMSF approach which approximates these models, is that CAT-PMSF permits the implementation of infinite mixture models that are not limited in their number of categories, and can therefore be expected to fit the data better.

**Fig. 1.**
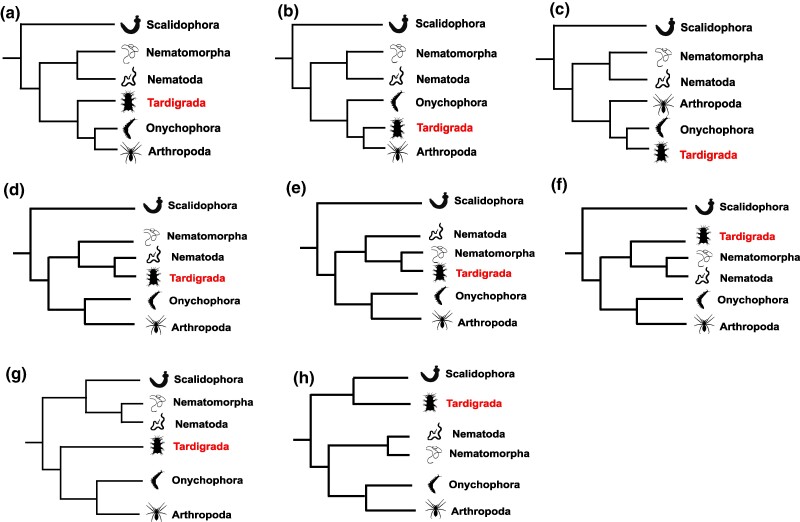
The eight topologies used to estimate CAT-Profiles to be exported using the PMSF procedure. The eight trees represent competing hypotheses of Ecdysozoan relationships. Three trees (a to c) display a monophyletic Nematoida within Cryptovermes, and differ for the placement of Tardigrada that is placed either as the sister of Arthropoda plus Onychophora (Lobopodia hypothesis), Onychophora (Protoarthropoda hypothesis), and Arthropoda (Tactopoda hypothesis). We then used three topologies (d to f) displaying Cryptovermes but with Tardigrada as a member of Nematoida (either as the sister of Nematoda, Nematomorpha, or Nematoida). Finally, we tested a tree (g) displaying Cycloneuralia (Nematoida sister of Scalidophora) and Lobopodia, and a tree (h) displaying Cryprovermes and Tardigrada as the sister of Scalidophora. As far as we can tell, the tree in (h) has never been proposed as a possible hypothesis of tardigrade relationships and we used it to test the effect of using a likely erroneous tree when estimating the CAT-Poisson site profile.

We used Parametric Bootstrap (see Materials and methods for details), the maximum likelihood equivalent of Bayesian posterior predictive resampling (Bollback 2002; Lartillot et al. 2007; Shepherd and Klaere 2019), to test the adequacy (e.g. Feuda et al. 2017; Giacomelli et al. 2022) of CAT-PMSF + G (hereafter CAT-PMSF) to our dataset. In addition, we tested the fit to the same data of other relevant models: LG + G (hereafter LG), Poisson-C60 + G (hereafter Poisson-C60), and LG-C60-PMSF + G (an implementation of the original PMSF procedure of Wang et al. [2018]; hereafter LG-C60-PMSF). Our parametric bootstrap analyses show that all the site-specific profiles generated with CAT-Poisson + G under the fixed topologies in Fig. 1 using Phylobayes (Lartillot et al. 2013) and exported to IQTree using the PMSF approach (CAT-PMSF), adequately describe the across-site compositional heterogeneity of our dataset. This is significant because other models tested failed to fit the data. CAT-PMSF supports Tardigrada as the sister of Arthropoda and Onychophora in a monophyletic Panarthropoda. This result is not recovered using LG, Poisson-C60, and LG-C60-PMSF, all of which support a sister group relationship between Tardigrada and Nematoda.

Our results demonstrate that CAT-PMSF significantly improves the modeling of across-site compositional heterogeneity under maximum likelihood and support the conclusion of Szánthó et al. (2023) that CAT-PMSF can break long-branch attraction artifacts, since Tardigrada, Nematoda, and Nematomorpha are long branched in our trees. Finally, our results provide further insights on the phylogenetic relationships of Ecdysozoa, resolving Tardigrada as the sister of Onychophora and Arthropoda (Lobopodia hypothesis) within Panarthropoda.

## Results

### Phylogenetic Inference Under CAT-PMSF Supports the Monophyly of Panarthropoda

**Fig. 2.**
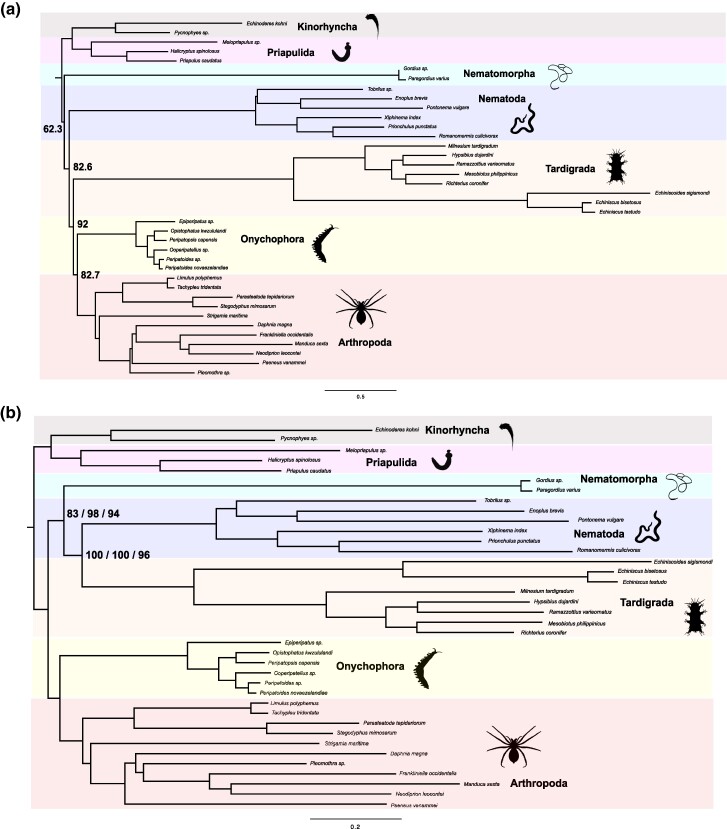
Results of the phylogenetic analyses. a) Majority rule consensus tree representing the results of all eight CAT-PMSF analyses. Ultrafast bootstrap support values have been calculated by averaging across the results of all analyses. b) Results were obtained using LG-C60-PMSF, LG, and Poisson-C60. From left to right, the bootstrap values for LG-C60-PMSF, LG, and Poisson-C60.

All CAT-PMSF analyses found support for the monophyly of Panarthropoda (Ultrafast Bootstrap—UFB ≥ 78, average UFB = 92; Fig. 2a, Table 1, and supplementary figs. S1 to S8, Supplementary Material online) irrespective of the topology used to infer the CAT-profile. In contrast, LG, Poisson-C60, and LG-C60-PMSF find support for Tardigrada as the sister of Nematoda (Fig. 2b, Table 1, and supplementary figs. S9 to S11, Supplementary Material online). All but one analysis support Cryptovermes (Nematoida sister to Panarthropoda; Fig. 2a and b, supplementary figs. S1 to S6 and S8 to S11, Supplementary Material online). However, the placement of Nematomorpha is unstable. Under LG, Poisson-C60, and LG-C60-PMSF, Tardigrada is always sister to Nematoda (UFB > 96; average UFB = 99; supplementary figs. S9 to S11, Supplementary Material online) and Nematomorpha is always the sister of these two lineages (UFB > 83; average UFB = 90.5; Fig. 2b, supplementary figs. S9 to S11, Supplementary Material online). Under CAT-PMSF, Nematoda is resolved as sister to Panarthropoda (UFB > 84; average UFB = 88.6), and Nematomorpha as the sister of both lineages (average UFB = 62.37; Fig. 2a, supplementary figs. S1 to S6 and S8, Supplementary Material online) in all but one analysis. The only analysis that does not support Cryptovermes recovers Nematomorpha as the sister to Scalidophora (Cephaloryncha hypothesis), but with low support (UFB = 64; supplementary fig. S7, Supplementary Material online). This result is obtained when the analysis is performed using the CAT-PMSF profile inferred using the fixed tree displaying Cycloneuralia (i.e. Nematoida sister to Scalidophora—Fig. 1g). Similarly, in all but one CAT-PMSF analysis, Tardigrada emerges as sister to Arthropoda plus Onychophora (Lobopodia hypothesis), Fig. 2a. The only exception is the CAT-PMSF analysis in which the model was inferred using the Protoarthropoda (Onychophora plus Tardigrada) tree, which recovered a tree displaying Protoarthropoda itself (supplementary fig. S2, Supplementary Material online). On average, Lobopodia emerges as the Panarthropoda variant with the highest support (UFB = 82.75—averaged across all eight CAT-PMSF analyses). Protoarthropoda is the second better-supported hypothesis with an average UFB = 12.75 (Table 1). None of the analyses found support for Tactopoda (Tardigrada sister to Arthropoda), average UFB (across all eight CAT-PMSF analyses) = 4.5.

**Table 1:** Ultrafast bootstrap support for key nodes as the model used to analyze the data is changed

		UFB support									
Model	Tree	Panarthropoda	Lobopodia	Protoarthopoda	Tactopoda	Nematoda plus Panarthropoda	Nematoda plus Tardigrada	Cryptovermes	Cephalorhyncha	Nematoida	Nematoida plus Tardigrada
LG	N/A	0	100	0	0	0	100	98	2	0	98
Poisson-C60	N/A	0	100	0	0	0	96	94	5	1	94
LG-C60-PMSF	1d	0	100	0	0	0	100	97	3	0	83
CAT-PMSF	1a	98	100	0	0	88	1	61	28	7	0
CAT-PMSF	1b	100	16	73	11	91	0	71	21	3	0
CAT-PMSF	1c	100	46	29	25	89	0	80	16	6	0
CAT-PMSF	1d	86	100	0	0	91	8	68	23	3	0
CAT-PMSF	1e	86	100	0	0	87	6	75	18	5	0
CAT-PMSF	1f	88	100	0	0	88	6	73	18	7	0
CAT-PMSF	1g	100	100	0	0	91	0	21	64	1	0
CAT-PMSF	1h	78	100	0	0	84	2	50	37	6	0

The tree topologies used to infer CAT-profiles are identified using the figure and panel legend where they are reported (e.g. Tree 1a is the tree in Fig. 1a).

### CAT-PMSF Describes the Compositional Heterogeneity of the Data Adequately

**Fig. 3.**
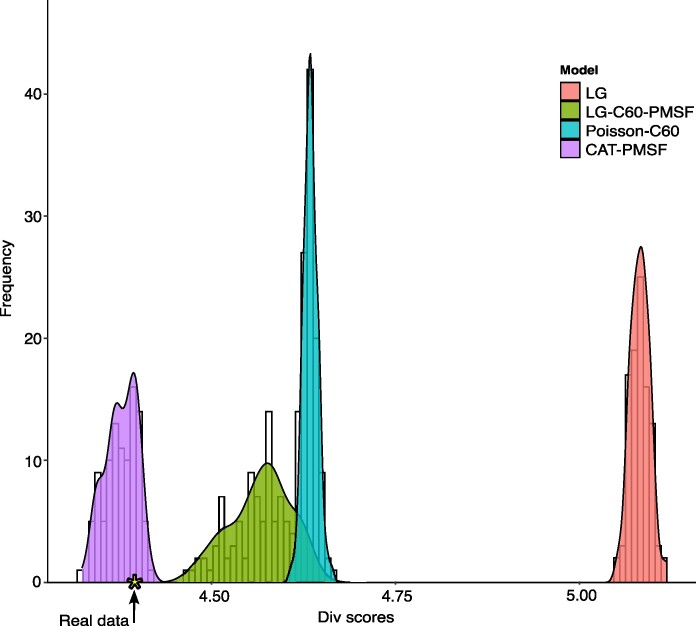
Distribution of *div* values for the simulated datasets under LG, Poisson-C60, LG-C60-PMSF, and one, representative, CAT-PMSF simulation. The star along the *x* axis indicates the *div* value of the real dataset. The *div* values for CAT-PMSF derive from the datasets simulated under topology 1A (Lobopodia).

We used parametric bootstrap (see Materials and methods for details) to test whether the models used in our study adequately described the data. The statistic we used (see Materials and methods for details) was “across-sites amino acid diversity” (*div*), the same statistic used in Phylobayes (see Phylobayes manual for details) to achieve the same goal using posterior predictive analysis (e.g. Feuda et al. 2017; Giacomelli et al. 2022 for applications). The results of the parametric bootstrap analyses are reported in Fig. 3 (distributions of *div* scores) and Table 2 (the *Z*-scores quantifying the difference between the compositional heterogeneity of the real data and that of simulated data). These results show that LG, Poisson-C60, and LG-C60-PMSF fail to fit the data (*Z*_LG_ = 52.87; *Z*_Poisson-C60_ = 25.37; *Z*_LG-C60-PMSF_ = 4.12). The average *Z*_CAT-PMSF_ score (average *Z*_CAT-PMSF_ = −1.13; see Table 2 for individual values) indicates that CAT-PMSF fits the data. The *div* score of the real data (*div* = 4.39) maps closely to the peaks of the distributions of *div* scores simulated under our eight CAT-PMSF models (Fig. 3). Differently, the *div* score of the real data does not fall within the distributions of *div* scores estimated from datasets simulated under LG, Poisson-C60, and LG-C60-PMSF (Fig. 3).

**Table 2:** Results of the model adequacy tests

Model	Fixed topology	Amino acid diversity			*Z*-score
		Real data	Simulated data		
			Average	SD	
LG	N/A	4.39	5.08	0.01	52.87
Poisson-C60	N/A	4.39	4.63	0.009	25.37
LG-C60-PMSF	1d	4.39	4.56	0.04	4.12
CAT-PMSF	1a	4.39	4.36	0.02	−0.89
CAT-PMSF	1b	4.39	4.36	0.018	−1.52
CAT-PMSF	1c	4.39	4.36	0.02	−1.22
CAT-PMSF	1d	4.39	4.37	0.02	−0.91
CAT-PMSF	1e	4.39	4.37	0.02	−1.11
CAT-PMSF	1f	4.39	4.36	0.02	−1.13
CAT-PMSF	1g	4.39	4.37	0.015	−1.31
CAT-PMSF	1h	4.39	4.37	0.02	−1.02

The fixed topologies used to estimate CAT-profiles are identified using the figure and panel legend where they are represented (e.g. Fixed topology 1a is the tree in Fig. 1a).

### Topology Tests Support Lobopodia as the Most Likely Resolution of Panarthropoda and Reject Cycloneuralia

Results of the Approximately Unbiased (AU) test (Table 3), performed under the CAT-PMSF models, show that only three of the tested topologies are never rejected (*P-*values ranging from 0.37 to 0.98). These three topologies recover Lobopodia but differ because of the way in which Nematoda and Nematomorpha are resolved. These three topologies resolve (i) Cryptovermes (monophyletic Nematoida sister to Panarthropoda), (ii) “alternative Cryptovermes” (with Nematoida paraphyletic—as in Fig. 2a), and (iii) Nematoda as sister to Panarthropoda and Nematomorpha as sister to Scalidophora (Cephalorhyncha hypothesis; supplementary fig. S7, Supplementary Material online). Table 3 also shows that the tree recovering Tardigrada as the sister to Scalidophora (Fig. 1h) is rejected under every CAT-PMSF model but the one where that same tree was used to infer the CAT-profile. The same is true for Cycloneuralia which is always rejected except when the test is performed using the CAT-PMSF profile inferred using the Cycloneuralia tree. All other hypotheses fall within these extremes being rejected under some, but not all CAT-PMSF models.

**Table 3:** Results of the approximately unbiased tests

	Tested topology										
Fixed topology	1a^a^	1b	1c	1d	1e	1f	1g	1h	2a^a^	S2	S7^a^
1a	0.35	0.02	0.02	0.02	0.02	0.02	0.000404	2.01E-27	0.6	0.03	0.6
1b	0.261	0.404	0.198	0.00095	0.000863	0.00094	0.000117	1.82E-77	0.342	0.705	0.478
1c	0.33	0.34	0.33	0.01	0.01	0.01	2.42E-07	9.15E-85	0.6	0.7	0.53
1d	0.31	0.00403	0.00419	0.0941	0.0944	0.0951	7.45E-05	3.02E-45	0.6	0.0096	0.604
1e	0.395	0.00373	0.00349	0.109	0.109	0.109	1.30E-06	1.06E-55	0.637	0.0047	0.549
1f	0.362	0.00454	0.00476	0.0913	0.0914	0.0926	5.93E-06	8.30E-76	0.636	0.00843	0.567
1g	0.37	0.0197	0.01	0.00479	0.00513	0.00495	0.13	0.00134	0.54	0.0219	0.694
1h	0.399	0.00056	0.00054	0.126	0.126	0.126	0.000924	0.0115	0.574	0.0028	0.604

Number in the cells are AU test *P*-values. Fixed topologies are the trees used to infer the CAT-PMSF model. Tested topologies are the trees that were tested using the AU test. Because each topology was tested eight times we used Bonferroni correction to set an appropriate level of significance (α = 0.00065). The Fixed topologies and the tested topologies are identified using the figure and panel legend where they are represented (e.g. Fixed and tested topology 1a is the tree in Fig. 1a, and tested topology S7 is the tree in supplementary fig. S7, Supplementary Material online).

^a^Identify tree topologies that are never rejected. Note that these topologies would not have been rejected even if we used a standard level of significance (α = 0.05), rather than the Bonferroni corrected significance level (see above). These are: Topology 1A—Lobopodia with monophyletic Nematoida sister of Lobopodia—“Cryptovermes”; Topology 2a Lobopodia with paraphyletic Nematoida (Nematoda sister of Panarthropoda and Nematomorpha sister of Panarthropoda plus Nematoda)—“Alternative Cryptovermes”; Topology S7 Lobopodia, Nematoda sister of Panarthropoda and Nematomorpha sister of Scalidophora—“Cephalorhyncha”.

## Discussion

Phylogenomics has so far failed to robustly resolve the position of Tardigrada, with previous studies having supported either an alliance with Nematoida (e.g. Dunn et al. 2008; Hejnol et al. 2009; Borner et al. 2014; Yoshida et al. 2017) or Panarthropoda (e.g. Campbell et al. 2011; Laumer et al. 2019; Howard et al. 2022). These two hypotheses are mutually exclusive and elicit not just competing genealogies for the ecdysozoan lineages but competing patterns of character evolution within Ecdysozoa.

Modeling heterogeneity is key to phylogenetic accuracy (e.g. Lartillot et al. 2007; Philippe et al. 2011; Puttick et al. 2018; Wang et al. 2019; Kapli et al. 2021; Tihelka et al. 2021; Giacomelli et al. 2022; Cai et al. 2024). Across-site compositionally heterogeneous models, such as CAT-Poisson and CAT-GTR provide for good modeling of across-site compositional heterogeneity (e.g. Lartillot et al. 2007; Kapli et al. 2021; Tihelka et al. 2021; Giacomelli et al. 2022; Cai et al. 2024), which cannot be achieved using standard across-site compositional homogeneous models (e.g. LG or GTR). Whelan and Halanych (2017) performed some simulations suggesting that across-site compositional heterogeneity can be modeled using partitioned models (where different across-site compositional homogeneous models are assigned to different genes or partitions of genes). While it is not the scope of this paper to address these models, we point out that, to our knowledge, no theoretical argument has been proposed that could explain the results of Whelan and Halanych (2017). This is because partitioned models accommodate across-genes, rather than across-sites heterogeneity. Indeed, the few empirical analyses that compared the fit of partitioned and across-site compositionally heterogeneous models on real datasets found partitioned models to fit across-site compositionally heterogeneous data significantly worse than across-site compositionally heterogeneous models (Feuda et al. 2017; Cai et al. 2024). As there is no reason to think that partitioned models, should, in general, fit across-site compositionally heterogeneous datasets, CXX-type models remain the only alternative to facilitate the description of across-site compositional heterogeneity in a maximum likelihood framework. However, these models are limited in the number of categories they can use, which has been shown to have the potential to lead to inadequate descriptions of the data (Giacomelli et al. 2022; Szánthó et al. 2023).

CAT-PMSF exports to maximum likelihood site-profiles of amino acid frequencies estimated using infinite mixture models (CAT-Poisson and CAT-GTR) in a Bayesian framework. These models are not limited in the number of categories that they use, and this has been shown to improve the modeling of across-site compositional heterogeneity in simulations and with real data (Giacomelli et al. 2022). Unfortunately, standard Bayesian analyses, where the CAT-based model is coestimated with the tree topology, are computationally challenging to practically intractable for large phylogenomic datasets (Wang et al. 2018; Szánthó et al. 2023—at the least with current Bayesian implementations). The PMSF procedure (Wang et al. 2018; Szánthó et al. 2023) allows the coestimation problem to be broken into two components that are solved sequentially. First, the CAT-based model (CAT-Poisson or CAT-GTR) is parameterized on a (user provided) fixed topology with Phylobayes. When the topology is fixed, parameterizing a CAT-Poisson or a CAT-GTR model is simpler, and good levels of convergence on the model parameters can be achieved in a reasonable amount of time for larger datasets. Once the CAT-based model is parameterized, it is exported to a format that can be read by IQTree, where cutting-edge implementations of fast algorithms for tree search and clade support estimation are available. By combining the best of current Bayesian and maximum likelihood approaches, CAT-PMSF has the potential to significantly improve the modeling of across-site compositional heterogeneous data. However, to our opinion, two important questions about CAT-PMSF remained unanswered. The first was whether this model improves the modeling of compositional heterogeneity enough to justify its use, given that CAT-PMSF is computationally more intense and time-consuming than the CXX-PMSF models of Wang et al. (2018). The second was whether fixing the topology when parametrizing the compositional profile might unduly affect its estimation. We answered both questions.

We developed a parametric bootstrap-based approach to test model adequacy in maximum likelihood, and we were able to show that CAT-PMSF significantly improves the modeling of across-site compositional heterogeneity. We compared the fit of CAT-PMSF to that of an across-site compositionally homogeneous model (LG) and two across-site compositionally heterogeneous models of different complexity (Poisson-C60 and LG-C60-PMSF). CAT-PMSF describes the data better than all the other approaches, being the only model that could adequately fit it. As is customary, we reported our results using *Z*-scores (Table 2). It is usually assumed that a model fits the data when its *Z*-score fall in the interval −2 < *Z*-score < 2. However, the interpretation of *Z*-scores depends on the shape of the distribution of *div* values, as we can only assume that 95% of data points fall in the −2 < *Z*-score < 2 interval if the data are normally distributed. Accordingly, we validated our results by plotting the *div* score obtained from the real data against the distributions of *div* scores calculated from simulated data (Fig. 3). For the CAT-PMSF models, we found the *div* score of the real data to map close to the peaks of the distributions of *div* scores from the simulated data. This strongly confirms the conclusion one would reach based exclusively on *Z*-scores (Table 2): CAT-PMSF fits the data.

*Z*-scores for LG, Poisson-C60, and LG-C60-PMSF suggest that all these models fail to fit the data (all *Z*-scores > 2). However, LG-C60-PMSF *Z*-score (Z_LG-C60-PMSF_ = 4.12) is much smaller than those calculated for Poisson-C60 (Z_Poisson-C60_ = 25.37) and LG (Z_LG_ = 52.87). This could be interpreted to suggest that LG-C60-PMSF is better at describing the across-site compositional heterogeneity of the data than LG and Poisson-C60. The distributions of *div* scores confirm the fit of LG to be worse than that of Poisson-C60 and LG-C60-PMSF. Differently, a comparison of the distributions of *div* scores for Poisson-C60 and LG-C60-PMSF (Fig. 3) shows that while Poisson-C60 does not fit as well as LG-C60-PMSF, the difference in fit between these models is overestimated when comparing their *Z*-scores only. The distribution of *div* values for LG-C60-PMSF is broader than that of Poisson-C60, and the Poisson-C60 distribution fully overlaps with the LG-C60-PMSF distribution. The Poisson-C60 distribution is located at the right end side of the LG-C60-PMSF distribution, where *div* scores indicating poorer fit are located. This confirms that Poisson-C60 does not fit as well as LG-C60-PMSF. However, while the LG-C60-PMSF distribution is skewed to the left of the Poisson-C60 distribution (toward *div* values indicating better fit), its peak is at the right end side, close to the peak of the Poisson-C60 distribution (Fig. 3). The proximity of the two peaks, at the right end of their overlapping distributions, indicates that the two models are comparably poor descriptors of the data. We conclude that to avoid misinterpreting the results of model adequacy tests, *Z*-scores are best interpreted in conjunction with an inspection of the distributions of *div* values from which they have been inferred.

We provide a tutorial (https://github.com/mgiacom/tardigrades_catpmsf) where a full description of the steps needed to perform a parametric bootstrap-based model adequacy test using IQTree is presented, together with a description of how *Z*-scores and distribution of *div* values are generated and interpreted.

The use of the poorly fitting LG, Poisson-C60, and LG-C60-PMSF resulted in the inference of the same topology, a tree where Nematoda, Nematomorpha, and Tardigrada (the three longest branches in the dataset) form a group (Fig. 2b). The well-fitting CAT-PMSF found a competing topology where the three long-branched taxa did not form a group (Fig. 2a), confirming that CAT-PMSF might have the ability to break attraction artifacts (Szánthó et al. 2023). CAT-PMSF analyses found Tardigrada to be a member of Panarthropoda, resolving the relationships of Tardigrada consistently with a diversity of other studies, both morphological and molecular (Budd 2001; Edgecombe 2010; Campbell et al. 2011; Legg et al. 2013; Yang et al. 2015; Laumer et al. 2019; Howard et al. 2022; Kihm et al. 2023).

CAT-PMSF-based phylogenetic analyses also reject Nematoida (Nematoda plus Nematomorpha), a clade of long-branched worms. In all but one analysis, Nematomorpha is recovered as sister to Arthropoda and Nematoda (i.e. Nematoida is paraphyletic within Cryptovermes; Fig. 2a). However, when the CAT-PMSF profile is inferred on a Cycloneuralia tree (Scalidophora plus Nematoida), Nematomorpha is recovered as sister to Scalidophora (supplementary fig. S7, Supplementary Material online), compatible with the Cephalorhyncha hypothesis. Some morphological support for Cephalorhyncha was provided by Adrianov and Malakhov (1995), though this was questioned by Schmidt-Rhaesa (1998). While Cephalorhyncha is a minority result with low support (UFB = 64) in our study, the fact that nematoid monophyly is invariably challenged by CAT-PMSF might suggest that this group may be artefactual. However, Nematomorpha has poor coverage in our dataset, and its instability might also be caused by their high proportion of missing data (70% *Gordius* and 71% *Paragordius*). Consistent with the second (high proportion of missing data) hypothesis, we note that in our AU tests, the tree displaying Cephalorhyncha (supplementary fig. S7, Supplementary Material online), Cryptovermes (Fig. 1a), and “alternative Cryptovermes”—the topology where Nematoida is a paraphyletic sister-grade to Panarthropoda, with Tardigrada sister to Onychophora plus Arthropoda (Fig. 2a)—cannot be distinguished.

Our results show that the fixed topology used to infer the CAT-profile might have a small biasing effect on the subsequent phylogenetic analyses. Table 1 shows that Prothoarthropoda (an alliance of Tardigrada and Onychophora) is only supported when the CAT-profile is inferred using a tree displaying Protoarthropoda. However, the support for Protoarthropoda is relatively low (UFB = 73), with support for alternative arrangements (Lobopodia and Tactopoda still being found—UFB = 16 and UFB = 11, respectively). Similarly, trees recovering Cycloneuralia and Tardigrada as sister to Scalidophora are rejected by the AU test (Table 3) under all CAT-PMSF models except the one inferred using, respectively, the Cycloneuralia or the Tardigrada plus Scalidophora trees. In both cases, the biasing effect is weaker than in the Protoarthropoda case, as Cycloneuralia and Tardigrada plus Scalidophora are never inferred in unconstrained CAT-PMSF analyses. Overall, when we look across all the CAT-PMSF trees, we find results to be largely independent of the topology used to infer the CAT-profile. Tardigrada is inferred as a member of Panarthropoda in all analyses (irrespective of the tree used to infer the CAT-profile), and Nematoida is never monophyletic (despite all CAT-Profiles have been inferred using trees assuming monophyletic Nematoida—supplementary figs. S1 to S8, Supplementary Material online). Furthermore, trees recovering Lobopodia are never rejected (Table 3) irrespective of the CAT-PMSF model used.

CAT-PMSF has significant potential, but we conclude that analyses are best repeated using different compositional profiles inferred under alternative trees. Results should be presented generating a consensus of results obtained from tree searches performed using different CAT-PMSF profiles (as we did in Fig. 2). When a tree is inferred by a CAT-PMSF analysis based on a compositional profile calculated using a tree displaying the same topology (as in the case of Protoarthropoda), the results should be considered with caution. However, when the same result is consistently inferred using multiple CAT-PMSF profiles that have been inferred using different fixed topologies, the result should be considered robust.

CAT-PMSF has only recently been developed (Szánthó et al. 2023) and, so far, only a handful of studies have applied it (e.g. Brabec et al. 2023; Mongiardino Koch et al. 2023; Cho et al. 2024; Redmond 2024). Of these studies, only Redmond (2024) attempted to test the fit of its CAT-PMSF model. To achieve this goal, Redmond (2024) used Phylobayes to test the fit of the CAT model from which the CAT-PMSF approximation was derived. This approach is not ideal because it assumes that the fit to the data to a CAT model and to its PMSF approximation are the same. While we expect the fit of CAT-based models and their PMSF approximation to be comparable, this hypothesis should be tested case by case. We developed a parametric bootstrap-based model adequacy test to evaluate the ability of CAT-PMSF (and other models) to adequately describe (i.e. fit; Shepherd and Klaere 2019) the across-site compositional heterogeneity of data in a maximum likelihood framework. Our results show that CAT-PMSF fits our dataset, differently from other considered models that fail to do so. This confirms that CAT-PMSF effectively translates the ability of CAT-profiles to model across-site compositional heterogeneity to maximum likelihood. Our parametric bootstrap-based model adequacy test will empower future studies to objectively test whether the model they are using adequately describes their data, providing a means to better discriminate among models and trees.

## Conclusions

CAT-PMSF improves the modeling of compositional heterogeneity in maximum likelihood, but the tree topology used to estimate the compositional profile may have a mild biasing effect. To control for that, analyses should be repeated using compositional profiles estimated on different tree topologies. Our results, based on analyses performed on multiple compositional profiles, strongly corroborate the view that Panarthropoda is a monophyletic group composed of tardigrades, onychophorans, and arthropods; the commonly recovered clade of tardigrades plus nematodes is likely a tree reconstruction artifact. Within Panarthropoda, our results favor Lobopodia (Tardigrada as the sister of Onychophora and Arthropoda). With reference to Nematoida, our results consistently support Nematoda as a member of Cryptovermes and the sister of Panarthropoda. The relationships of Nematomorpha are unstable but this might reflect a limitation of our dataset, in which Nematomorpha are poorly represented. Most of our analyses find Nematomorpha as a member of Cryptovermes but we cannot reject Nematomorpha as sister to Scalidophora (Cephalorhyncha hypothesis). In any case, Cycloneuralia (an alliance of Nematoida and Scalidophora usually recovered using morphological data) seems rejected.

## Materials and Methods

### Orthology Inference and Matrix Assembly

We used the BUSCO (Simão et al. 2015) to build our matrix. The core set of 971 metazoan BUSCO genes was searched and extracted from the proteomes of 38 ecdysozoans and two outgroups (downloaded from NCBI). We initially ran the BUSCO analysis for each proteome, retrieving all the homologs—orthologues and paralogues when more than one hit was found. Of the 971 initial families, 52 did not contain tardigrades or nematodes and were excluded. The 919 remaining families were aligned with MAFFT 7.3.8 (*-linsi*; Katoh and Standley 2013) and gappy columns were trimmed with trimAL 1.2 (*-gappyout*; Capella-Gutiérrez et al. 2009). A further cleaning step was performed using Al2Phylo (*-m 50 -p 0.25*; Ballesteros and Hormiga 2016) to remove sequences containing >25% of gaps and keeping alignments with more than 50 amino acid positions. Gene trees were built for each BUSCO family (i.e. each retained orthogroup/homolog set) with IQTree v.2 (Minh et al. 2020) under the best-fitting model selected by *Modelfinder* (Kalyaanamoorthy et al. 2017)—including site-heterogeneous mixture models with fixed number of categories (CXX models; Si Quang et al. 2008). BUSCO genes, in which one or more of the taxa had more than one hit, were investigated to decide which paralogue to retain if any. If the paralogues formed a monophyletic group (i.e. if they were *in-paralogues*), the copy with the shortest branch length was retained. If the paralogs did not form a monophyletic group (i.e. they were *out-paralogues*), the gene family was excluded to minimize the inclusion of hidden paralogues (sensu Siu-Ting et al. 2019; Mulhair et al. 2022; Pisani et al. 2022; McCarthy et al. 2023) in the final dataset. The final set of retained gene families was realigned and curated using the same steps, MAFFT, trimAL, and Al2Phylo. New gene trees were built (same approach described above) and further cleaned to remove unusually long branches that might negatively affect downstream analyses, using a bespoke script (https://github.com/mgiacom/tardigrades_catpmsf). Unusually long branches in a putative single-copy gene tree can represent hidden paralogues, sequences with an unusually high proportion of missing data, contaminants, sequences with very high evolutionary rates, or strong compositional heterogeneity. In all such cases, the inclusion of these sequences has the potential to mislead downstream analyses. We removed all sequences associated with branches with a length >2 SDs above the mean for the considered gene family (Lozano-Fernandez et al. 2016). We found, as expected, that many of the tardigrade and nematode sequences had unusually long branches. Removal of sequences with long branches was repeated two times because the presence of sequences with extremely long branches can artificially skew mean and SD calculations, hiding other sequences that would otherwise be identified as long branched. Our final dataset included 571 BUSCO genes (70,088 amino acid positions) and 29.6% missing data, and it is available in our GitHub repository (https://github.com/mgiacom/tardigrades_catpmsf).

### Site-Specific Amino Acids Profiles Estimation

The CAT-PMSF approach (Szánthó et al. 2023) uses Bayesian analyses to infer site-specific amino acid frequency profiles estimated using infinite mixture models (CAT-Poisson or CAT-GTR), under a fixed topology. These profiles are then exported to a format that can be read by IQTree (Minh et al. 2020), where they are used to perform ML tree searches.

We inferred compositional profiles under CAT-Poisson (Lartillot and Philippe 2004) in Phylobayes v1.9 (Lartillot et al. 2013). Because CAT-PMSF profiles are inferred using a fixed topology to improve convergence (Szánthó et al. 2023), we generated eight site-profiles assuming different tree topologies (Fig. 1) that account for taxonomic uncertainty in ecdysozoan relationships. Two independent chains were run to estimate each site-profile (for a total of 16 runs) and convergence was assessed using *tracecomp*, ensuring that the Effective Sample Size was >100 and that the relative difference—*reldiff* < 0.3 for all parameters. The site-profiles were extracted using the *readpb -ss* command, sampling 100 points of the posterior parameter space. These site-profiles were converted in a format readable by IQTree using the script *convert_site.py*, see Szánthó et al. (2023) for details.

### Maximum Likelihood Phylogenetic Inference and Topology Testing

Unconstrained phylogenetic inferences were performed in IQtree v.2 (Minh et al. 2020), combining the Poisson exchangeability matrix (Felsenstein 1981) and a discrete gamma rate model with four categories (Yang 1994) with the eight site-profiles (*-fs*) estimated using Phylobayes (i.e. using eight CAT-PMSF models). Unconstrained phylogenetic analyses were also performed with the LG, Poisson-C60, and the original PMSF method of Wang et al. (2018), which we used to approximate LG-C60 (LG-C60-PMSF). As pointed out above, all models used a Gamma distribution to account for across-site rate variation. Support values were estimated using the Ultrafast Bootstraps (UFB; *-bb 1000*; Hoang et al. 2018).

Hypothesis testing was performed to investigate whether alternative hypotheses of ecdysozoan relationships could be rejected by performing AU tests (Shimodaira 2002) with 10,000 replicates in IQtree v.2. The AU tests compared 11 trees: the eight trees used to estimate the CAT-PMSF models and the three distinguished topologies that emerged from the eight CAT-PMSF analyses. The AU tests were performed under each of the eight CAT-PMSF models. Accordingly, each tree topology was tested eight times. We used the Bonferroni correction to take into consideration that each topology was tested multiple times (Bonferroni corrected significance values *α* = 0.00625).

### Parametric Bootstrap Test of Model Adequacy

Parametric bootstrap is a statistical method that can be used to evaluate the performance of a model in describing the data (= testing model adequacy) under maximum likelihood. Parametric bootstrap should not be confused with standard (nonparametric) bootstrap, which is used to estimate support for clades in a tree. To perform a parametric bootstrap analysis, datasets are simulated (of the same size of the original dataset) under the model used to analyze the data. It is fundamental that the model used to simulate the data is parametrized exactly as the one used to analyze the data. A defined number of datasets is simulated (e.g. 100 datasets), and a statistic of interest is measured from each simulated dataset to generate a distribution of values. After that, the same statistic is calculated for the original dataset. If the values observed for the original data fall within the distribution of the simulated datasets, the model is said to adequately describe (or fit) the data. If the value calculated for the original data does not fall within the distribution of values generated from the simulated data, the model does not adequately describe (or fit) the data. Deviations of real values from the average of the distribution are usually expressed using standard deviates (*Z*-scores). Phylogenetic model adequacy is more frequently tested in a Bayesian framework, using posterior predictive analysis (Lartillot et al. 2007; Morgan et al. 2013; Tarver et al. 2016; Feuda et al. 2017; Williams et al. 2020; Giacomelli et al. 2022; Mulhair et al. 2022; Cai et al. 2024), the Bayesian equivalent of Parametric Bootstrap. However, given the current tendency for the development of complex models in maximum likelihood (e.g. the CAT-PMSF models; Szánthó et al. 2023), it is important that we develop tools to test the fit of models also in a maximum likelihood framework. This is what we did, and we provide a tutorial, code, and scripts to test model adequacy using parametric bootstrap (https://github.com/mgiacom/tardigrades_catpmsf).

CAT-PMSF is a modification of the PMSF approach of Wang et al. (2018) and, as in the case of standard PMSF, testing its relative fit (Shepherd and Klaere 2019) against that of other models using standard model-fit methods (e.g. the Bayesian Information Criterion or the Akaike Information Criterion) is not possible (see Wang et al. 2018; Redmond 2024) and discussion. However, model adequacy tests (e.g. Bollback 2002; Lemmon and Moriarty 2004; Höhna et al. 2018; Shepherd and Klaere 2019; Fabreti et al. 2024) can be used to test whether CAT-PMSF can adequately describe (i.e. fit) the data (Shepherd and Klaere 2019), with reference to across-site compositional heterogeneity. Under maximum likelihood, model adequacy tests can be implemented by simulating data using a parametric bootstrap (see above). The statistics we chose to test the ability of CAT-PMSF (and of other relevant models) to describe the across-site compositional heterogeneity of the data is “across-sites amino acid diversity” (*div—*the average number of amino acids observed across the sites of a dataset). This is the same statistics used in posterior predictive tests of the fit of across-site compositional heterogeneity in Phylobayes (see the Phylobayes manual).

We followed Morgan et al. (2013), Tarver et al. (2016), Feuda et al. (2017), Puttick et al. (2018), Williams et al. (2020), Giacomelli et al. (2022), Mulhair et al. (2022), and Cai et al. (2024) and expressed the deviation of the *div* score of real data from that of simulated data using standard deviates (*Z*-scores). However, we also plotted *div* scores from simulated datasets to visualize their distributions. We used our pipeline to estimate the fit of LG, Poisson-C60, LG-C60-PMSF, and our eight CAT-PMSF models. However, we only plotted the distributions of *div* scores for LG, Poisson-C60, LG-C60-PMSF, and one exemplar CAT-PMSF model (the one inferred using the Lobopodia tree in Fig. 1).

We used AliSim (Ly-Trong et al. 2022, 2023) in IQTree to simulate (using the parametric bootstrap) 100 datasets under LG, Poisson-C60, LG-C60-PMSF, and CAT-PMSF (all eight models). Simulating datasets under PMSF is not straightforward and we developed a bespoke Python script to generate nexus files to be used with IQTree, simulating (using the PMSF models) sites one at a time (https://github.com/mgiacom/tardigrades_catpmsf). The simulated sites are then concatenated to generate simulated alignments. We assumed that a model adequately described the data when its *Z*-score fell in the interval −2 < *Z*-score < 2 (Feuda et al. 2017; Giacomelli et al. 2022). *Z*-scores progressively larger than 2 or smaller than −2 indicate a progressively worse fit of the model to the data, with *Z* > 10 or *Z* < −10 indicating a very poor fit (Giacomelli et al. 2022). A *Z*-score falling within the −2 < *Z*-score < 2 interval certainly identifies a model that fits the data only when the distribution of simulated *div* scores is normal. When that is not the case, distributions of simulated *div* scores (see above) must be plotted to ensure that *Z*-scores reflect our expectations. Our interpretations of *Z*-scores were, therefore, grounded on the visualization of the distributions of simulated *div* values and their distance to the real data *div* score. Following the convention for the implementation of model adequacy tests in Phylobayes, we express *Z*-scores as positive values when the model underestimates across-sites amino acid diversity and as negative values when the model overestimates diversity. This implies that the *Z*-scores presented in our paper (exactly as those generated by Phylobayes) are negatives of the calculated *Z*-scores.

## Supplementary Material

evae273_Supplementary_Data

## Data Availability

All data used were obtained from public data repositories. Our datasets, scripts, and tutorials are publicly available at https://github.com/mgiacom/tardigrades_catpmsf.
