# Supplementary material for: CAT-Posterior Mean Site Frequencies Improves Phylogenetic Modeling Under Maximum Likelihood and Resolves Tardigrada as the Sister of Arthropoda Plus Onychophora
Source: Genome Biol Evol. 2024 Dec 23;17(1):evae273. doi: 10.1093/gbe/evae273 (PMC11756273; doi:10.1093/gbe/evae273)
Supplement: evae273_Supplementary_Data [file evae273_supplementary_data.pdf]

CAT-PMSF improves phylogenetic modelling under maximum likelihood and resolves Tardigrada within Panarthropoda, as the sister of Arthropoda plus Onychophora

Supplementary information

Mattia Giacomelli, Matteo Vecchi, Roberto Guidetti, Lorena Rebecchi, Philip C.J. Donoghue, Jesus Lozano-Fernandez, Davide Pisani

Figure S1. Tree inferred from the CAT-PMSF analyses using the compositional profile inferred under the fixed topology of Fig. 1A.

Figure S2. Tree inferred from the CAT-PMSF analyses using the compositional profile inferred under the fixed topology of Fig. 1B.

Figure S3. Tree inferred from the CAT-PMSF analyses using the compositional profile inferred under the fixed topology of Fig. 1C.

Figure S4. Tree inferred from the CAT-PMSF analyses using the compositional profile inferred under the fixed topology of Fig. 1D.

Figure S5. Tree inferred from the CAT-PMSF analyses using the compositional profile inferred under the fixed topology of Fig. 1E.

Figure S6. Tree inferred from the CAT-PMSF analyses using the compositional profile inferred under the fixed topology of Fig. 1F.

Figure S7. Tree inferred from the CAT-PMSF analyses using the compositional profile inferred under the fixed topology of Fig. 1G.

Figure S8. Tree inferred from the CAT-PMSF analyses using the compositional profile inferred under the fixed topology of Fig. 1H.

Figure S9. Tree inferred from the analysis performed under LG.

Figure S10. Tree inferred from the analysis performed under Poisson-C60.

Figure S11. Tree inferred from the analysis performed using LG-C60-PMSF.

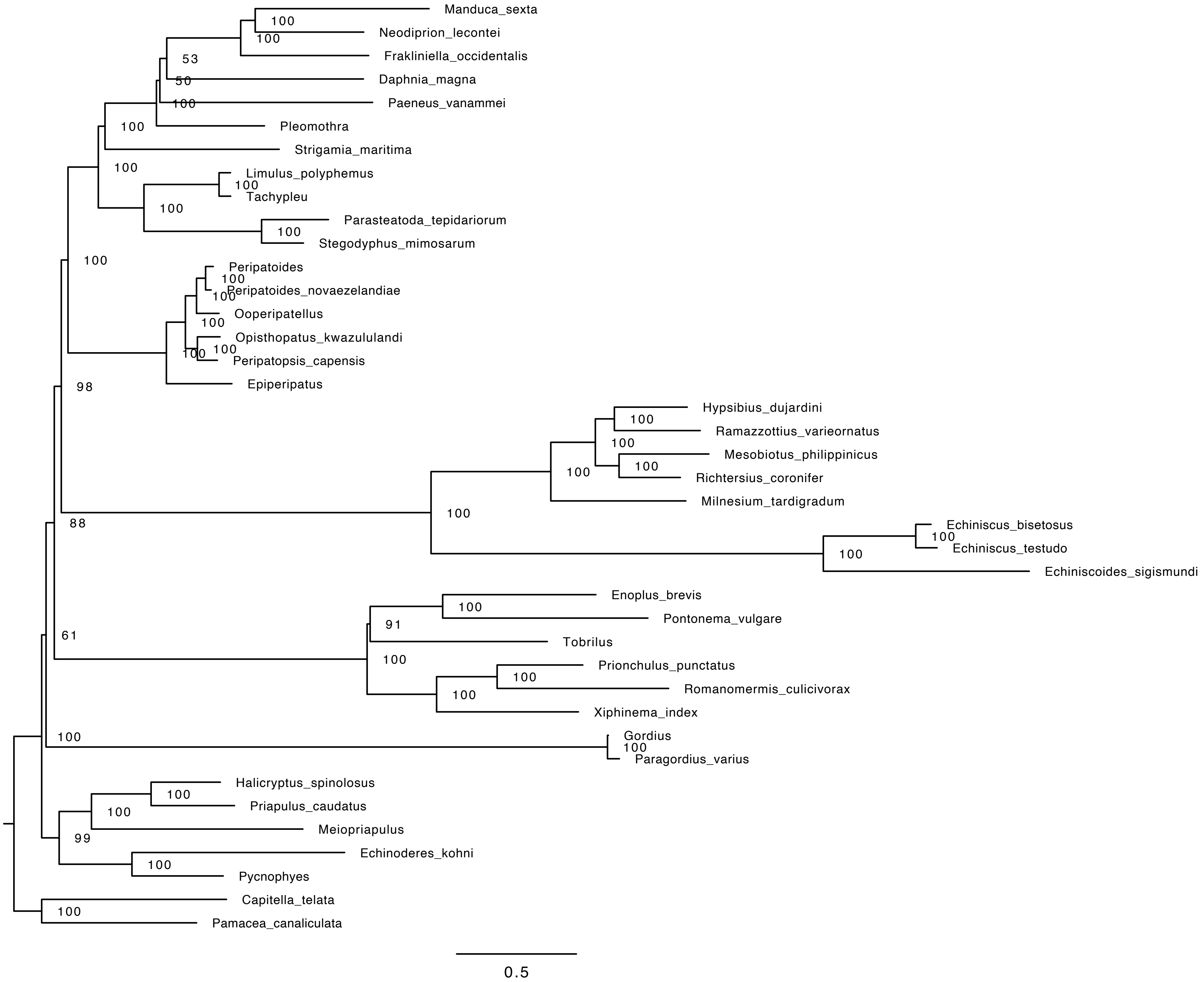

Manduca_sexta
Neodiprion_lecontei
Frakliniella_occidentalis
Daphnia_magna
Paeneus_vanammei
Pleomothra
Strigamia_maritima
Limulus_polyphemus
Tachypleu
Parasteatoda_tepidariorum
Stegodyphus_mimosarum
Peripatoides
Peripatoides_novaezelandiae
Ooperipatellus
Opisthopatus_kwazululandi
Peripatopsis_capensis
Epiperipatus
Hypsibius_dujardini
Ramazzottius_varieornatus
Mesobiotus_philippinicus
Richtersius_coronifer
Milnesium_tardigradum
Echiniscus_bisetosus
Echiniscus_testudo
Echiniscoides_sigismundi
Enoplus_brevis
Pontonema_vulgare
Tobrilus
Prionchulus_punctatus
Romanomermis_culicivorax
Xiphinema_index
Gordius
Paragordius_varius
Halicryptus_spinolosus
Priapulus_caudatus
Meiopriapulus
Echinoderes_kohni
Pycnophyes
Capitella_telata
Pamacea_canaliculata
100
100
53
50
100
100
100
100
100
100
100
100
100
100
100
98
100
100
100
100
100
100
88
100
91
61
100
100
100
100
100
100
100
99
100
100
0.5

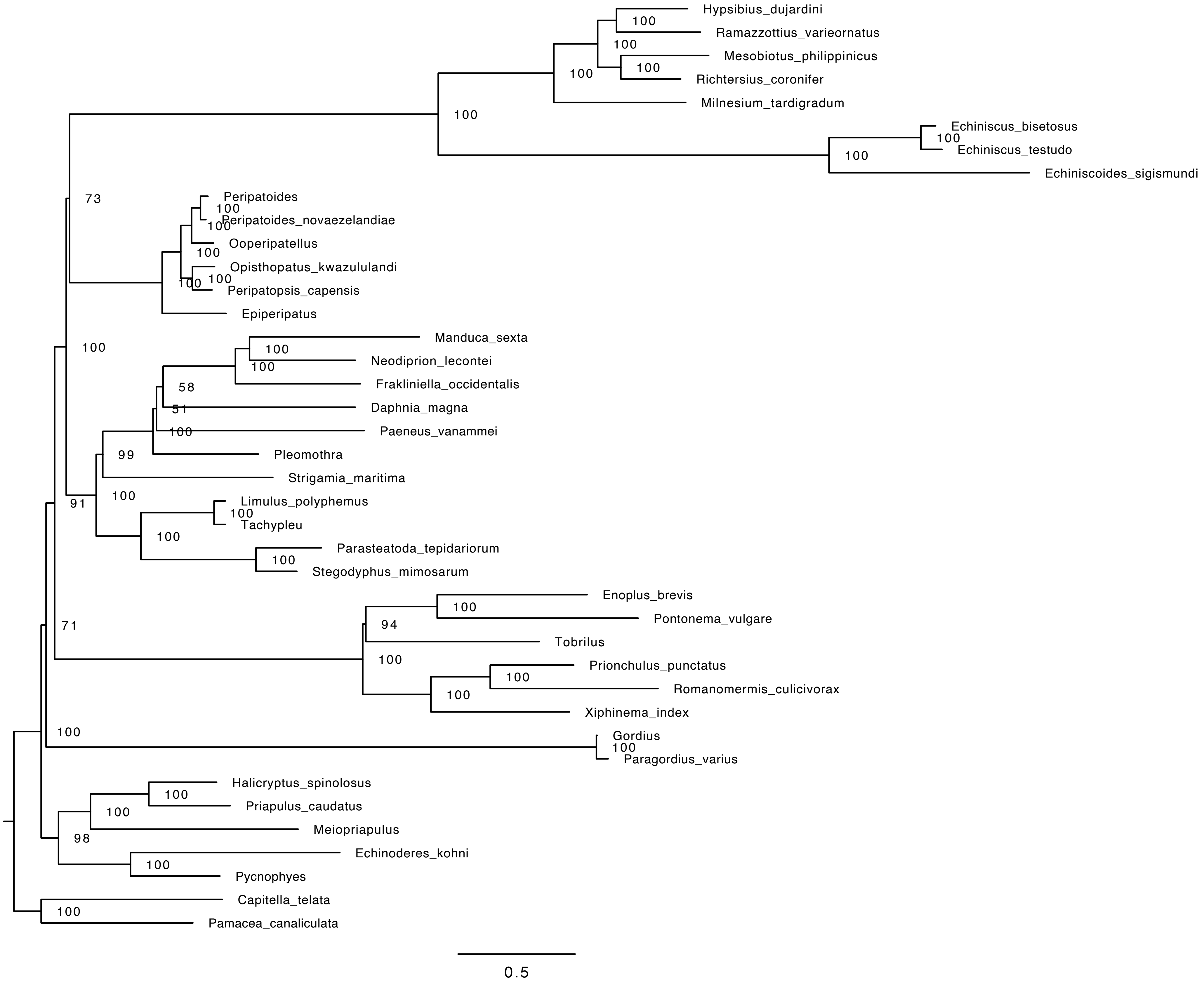

Hypsibius_dujardini
Ramazzottius_varieornatus
Mesobiotus_philippinicus
Richtersius_coronifer
Milnesium_tardigradum
Echiniscus_bisetosus
Echiniscus_testudo
Echiniscoides_sigismundi
Peripatoides
Peripatoides_novaezelandiae
Ooperipatellus
Opisthopatus_kwazululandi
Peripatopsis_capensis
Epiperipatus
Manduca_sexta
Neodiprion_lecontei
Frakliniella_occidentalis
Daphnia_magna
Paeneus_vanammei
Pleomothra
Strigamia_maritima
Limulus_polyphemus
Tachypleu
Parasteatoda_tepidariorum
Stegodyphus_mimosarum
Enoplus_brevis
Pontonema_vulgare
Tobrilus
Prionchulus_punctatus
Romanomermis_culicivorax
Xiphinema_index
Gordius
Paragordius_varius
Halicryptus_spinolosus
Priapulus_caudatus
Meiopriapulus
Echinoderes_kohni
Pycnophyes
Capitella_telata
Pamacea_canaliculata
100
73
71
91
99
58
51
94
98
0.5

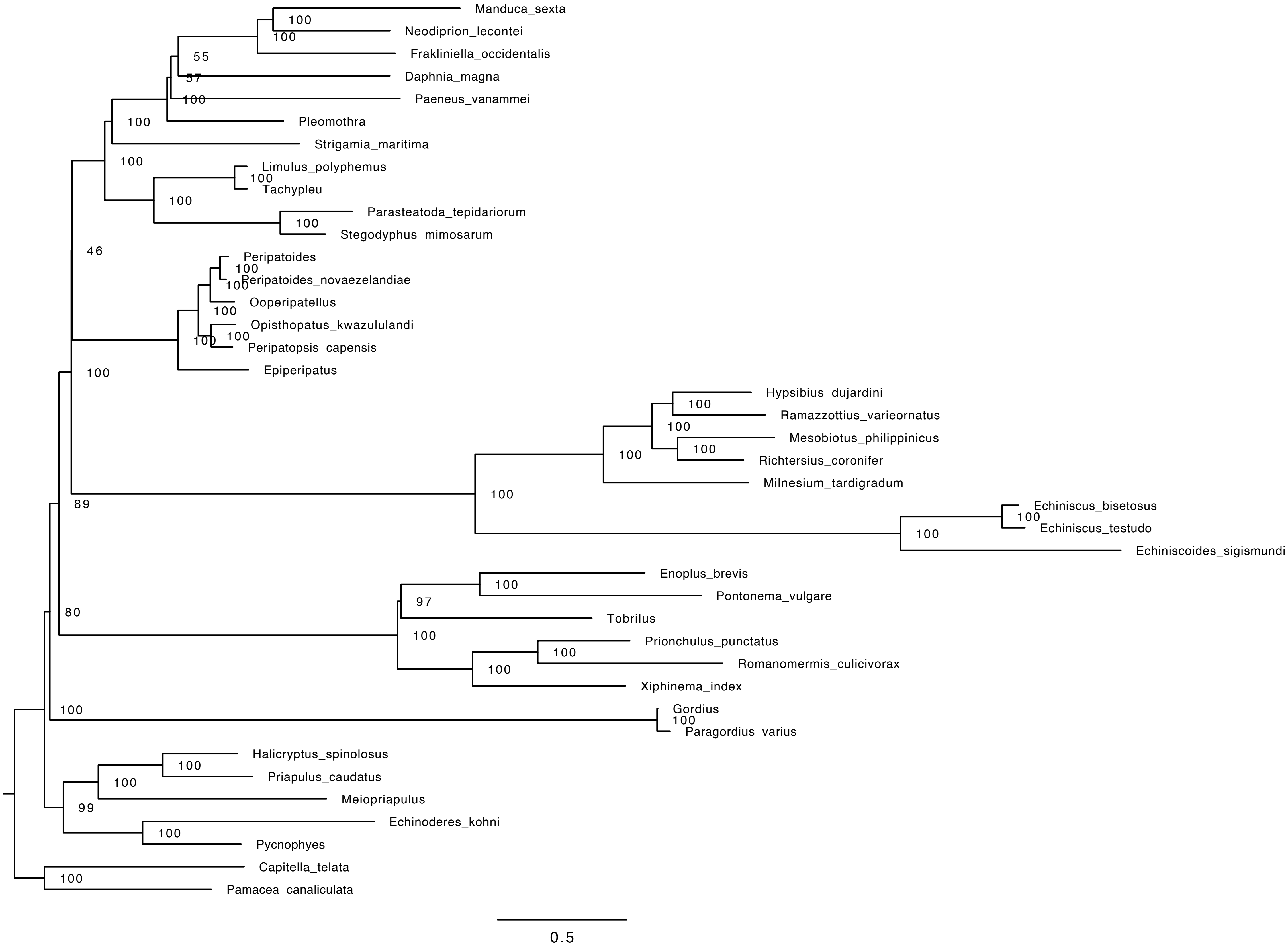
Manduca_sexta
Neodiprion_lecontei
Frakliniella_occidentalis
Daphnia_magna
Paeneus_vanammei
Pleomothra
Strigamia_maritima
Limulus_polyphemus
Tachypleu
Parasteatoda_tepidariorum
Stegodyphus_mimosarum
Peripatoides
Peripatoides_novaezelandiae
Ooperipatellus
Opisthopatus_kwazululandi
Peripatopsis_capensis
Epiperipatus
Hypsibius_dujardini
Ramazzottius_varieornatus
Mesobiotus_philippinicus
Richtersius_coronifer
Milnesium_tardigradum
Echiniscus_bisetosus
Echiniscus_testudo
Echiniscoides_sigismundi
Enoplus_brevis
Pontonema_vulgare
Tobrilus
Prionchulus_punctatus
Romanomermis_culicivorax
Xiphinema_index
Gordius
Paragordius_varius
Halicryptus_spinolosus
Priapulus_caudatus
Meiopriapulus
Echinoderes_kohni
Pycnophyes
Capitella_telata
Pamacea_canaliculata
100
55
57
46
89
80
97
99
0.5

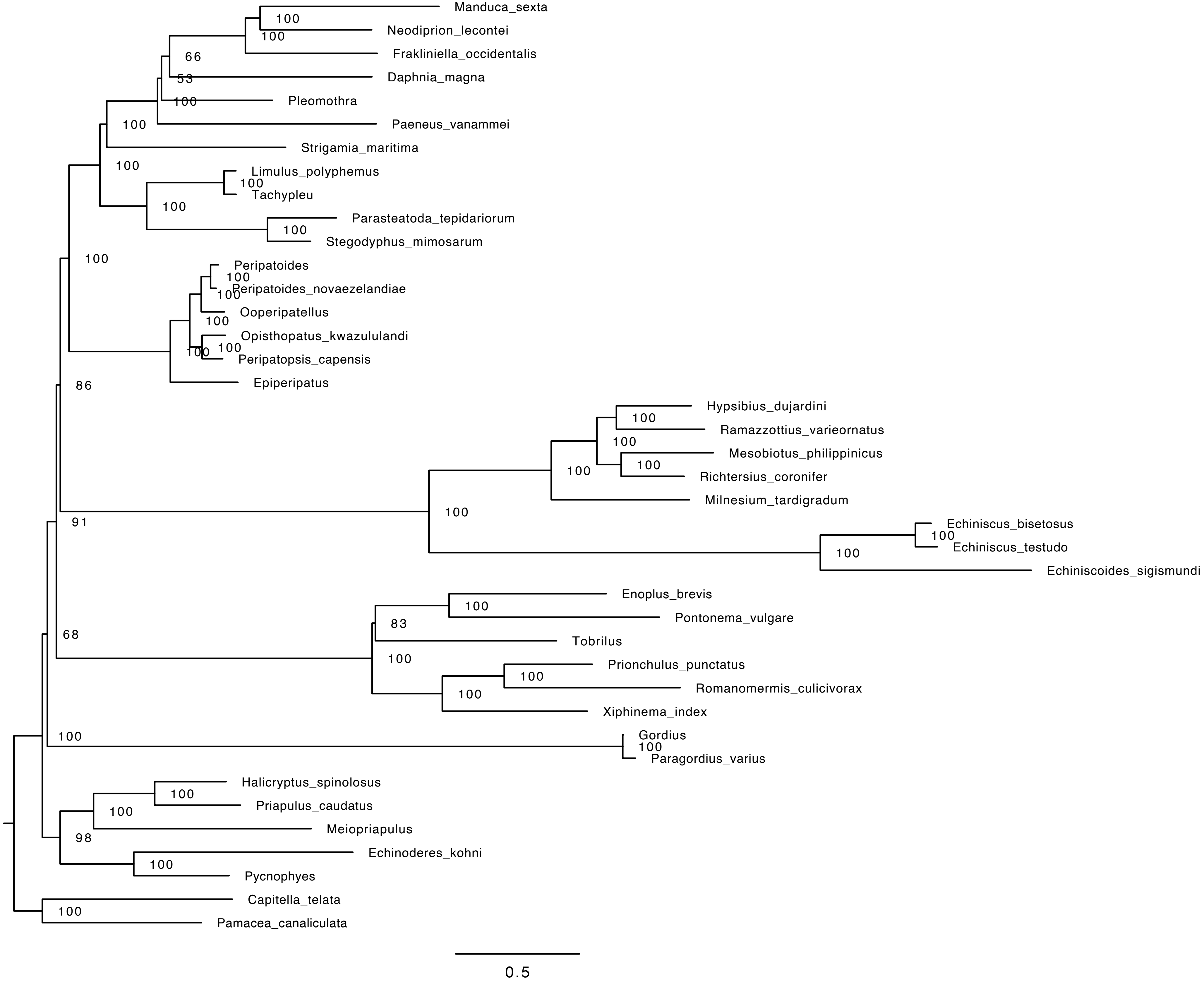

Manduca_sexta
Neodiprion_lecontei
Frakliniella_occidentalis
Daphnia_magna
Pleomothra
Paeneus_vanammei
Strigamia_maritima
Limulus_polyphemus
Tachypleu
Parasteatoda_tepidariorum
Stegodyphus_mimosarum
Peripatoides
Peripatoides_novaezelandiae
Ooperipatellus
Opisthopatus_kwazululandi
Peripatopsis_capensis
Epiperipatus
Hypsibius_dujardini
Ramazzottius_varieornatus
Mesobiotus_philippinicus
Richtersius_coronifer
Milnesium_tardigradum
Echiniscus_bisetosus
Echiniscus_testudo
Echiniscoides_sigismundi
Enoplus_brevis
Pontonema_vulgare
Tobrilus
Prionchulus_punctatus
Romanomermis_culicivorax
Xiphinema_index
Gordius
Paragordius_varius
Halicryptus_spinolosus
Priapulus_caudatus
Meiopriapulus
Echinoderes_kohni
Pycnophyes
Capitella_telata
Pamacea_canaliculata
0.5

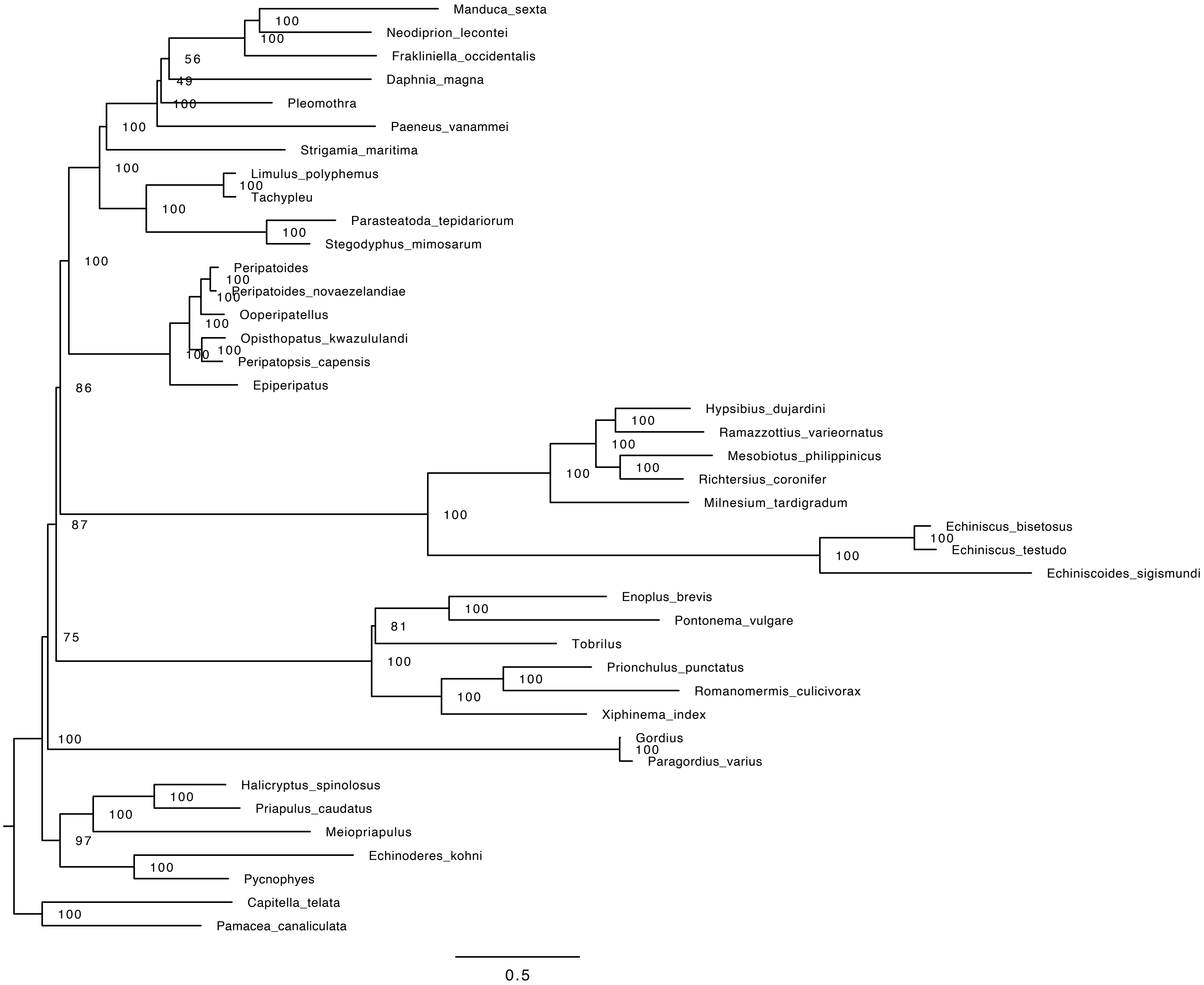
Manduca_sexta
100
Neodiprion_lecontei
100
Frakliniella_occidentalis
56
Daphnia_magna
49
Pleomothra
100
Paeneus_vanammei
100
Strigamia_maritima
100
Limulus_polyphemus
100
Tachypleu
100
Parasteatoda_tepidariorum
100
Stegodyphus_mimosarum
100
Peripatoides
100
Peripatoides_novaezelandiae
100
Ooperipatellus
100
Opisthopatus_kwazululandi
100
Peripatopsis_capensis
100
Epiperipatus
86
Hypsibius_dujardini
100
Ramazzottius_varieornatus
100
Mesobiotus_philippinicus
100
Richtersius_coronifer
100
Milnesium_tardigradum
100
Echiniscus_bisetosus
100
Echiniscus_testudo
100
Echiniscoides_sigismundi
87
Enoplus_brevis
100
Pontonema_vulgare
81
Tobrilus
100
Prionchulus_punctatus
100
Romanomermis_culicivorax
100
Xiphinema_index
75
100
Gordius
100
Paragordius_varius
Halicryptus_spinolosus
100
Priapulus_caudatus
100
Meiopriapulus
97
Echinoderes_kohni
100
Pycnophyes
Capitella_telata
100
Pamacea_canaliculata
0.5

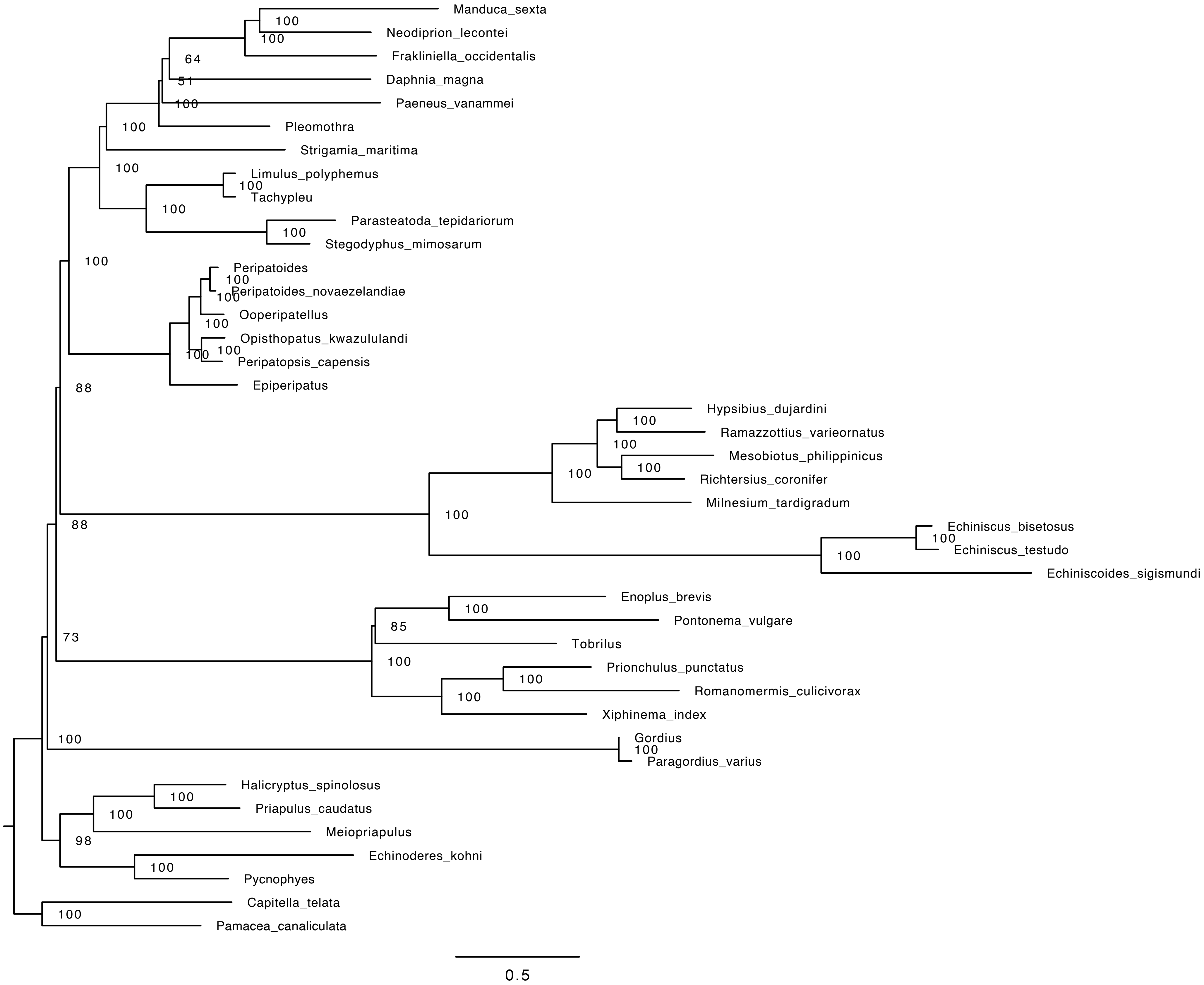
Manduca_sexta
100
Neodiprion_lecontei
100
Frakliniella_occidentalis
64
Daphnia_magna
51
Paeneus_vanammei
100
Pleomothra
100
Strigamia_maritima
100
Limulus_polyphemus
100
Tachypleu
100
Parasteatoda_tepidariorum
100
Stegodyphus_mimosarum
100
Peripatoides
100
Peripatoides_novaezelandiae
100
Ooperipatellus
100
Opisthopatus_kwazululandi
100
Peripatopsis_capensis
100
Epiperipatus
88
Hypsibius_dujardini
100
Ramazzottius_varieornatus
100
Mesobiotus_philippinicus
100
Richtersius_coronifer
100
Milnesium_tardigradum
100
Echiniscus_bisetosus
100
Echiniscus_testudo
100
Echiniscoides_sigismundi
88
Enoplus_brevis
100
Pontonema_vulgare
85
Tobrilus
100
Prionchulus_punctatus
100
Romanomermis_culicivorax
100
Xiphinema_index
73
100
Gordius
100
Paragordius_varius
Halicryptus_spinolosus
100
Priapulus_caudatus
100
Meiopriapulus
98
Echinoderes_kohni
100
Pycnophyes
Capitella_telata
100
Pamacea_canaliculata
0.5

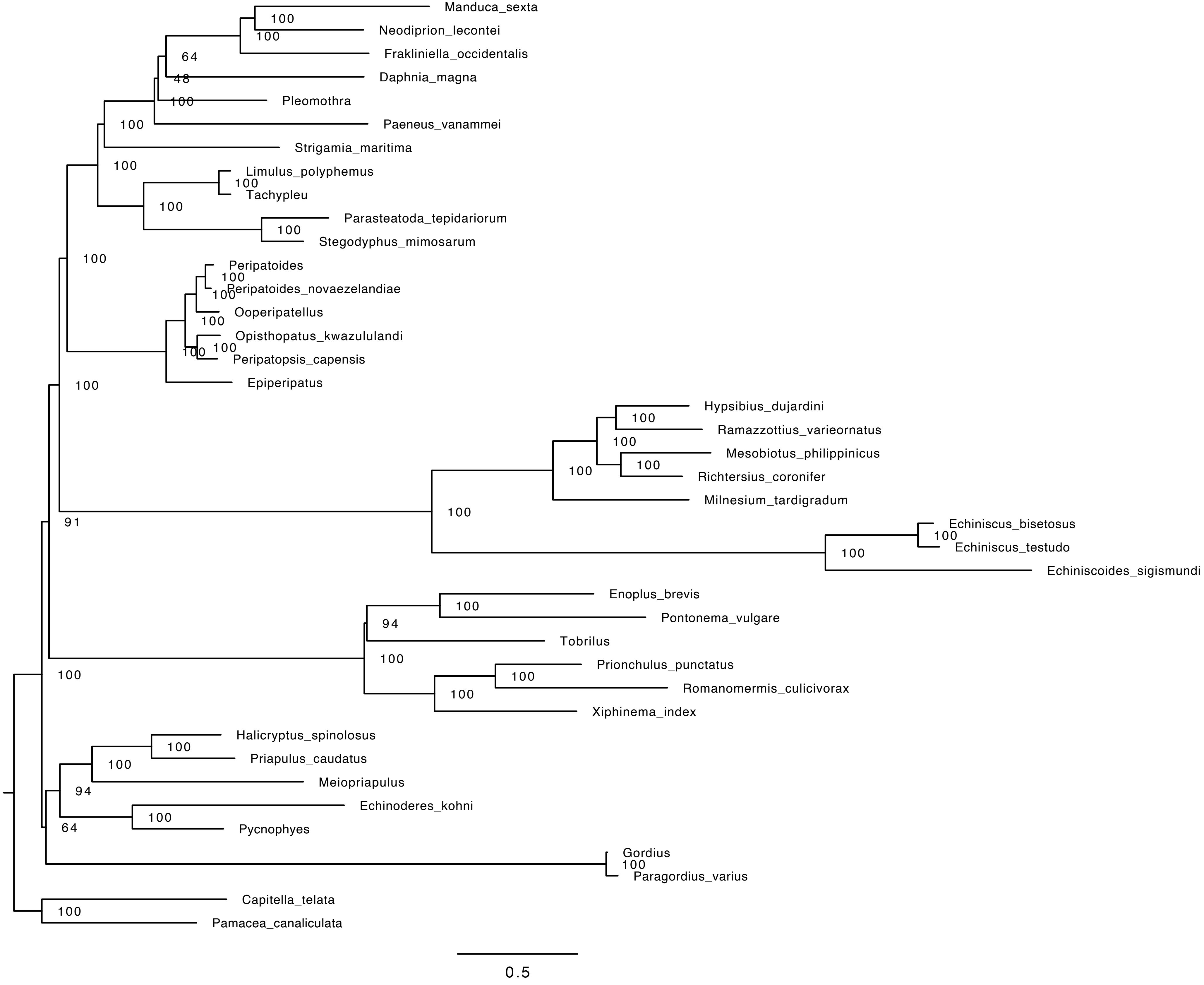

Manduca_sexta
Neodiprion_lecontei
Frakliniella_occidentalis
Daphnia_magna
Pleomothra
Paeneus_vanammei
Strigamia_maritima
Limulus_polyphemus
Tachypleu
Parasteatoda_tepidariorum
Stegodyphus_mimosarum
Peripatoides
Peripatoides_novaezelandiae
Ooperipatellus
Opisthopatus_kwazululandi
Peripatopsis_capensis
Epiperipatus
Hypsibius_dujardini
Ramazzottius_varieornatus
Mesobiotus_philippinicus
Richtersius_coronifer
Milnesium_tardigradum
Echiniscus_bisetosus
Echiniscus_testudo
Echiniscoides_sigismundi
Enoplus_brevis
Pontonema_vulgare
Tobrilus
Prionchulus_punctatus
Romanomermis_culicivorax
Xiphinema_index
Halicryptus_spinolosus
Priapulus_caudatus
Meiopriapulus
Echinoderes_kohni
Pycnophyes
Gordius
Paragordius_varius
Capitella_telata
Pamacea_canaliculata
0.5

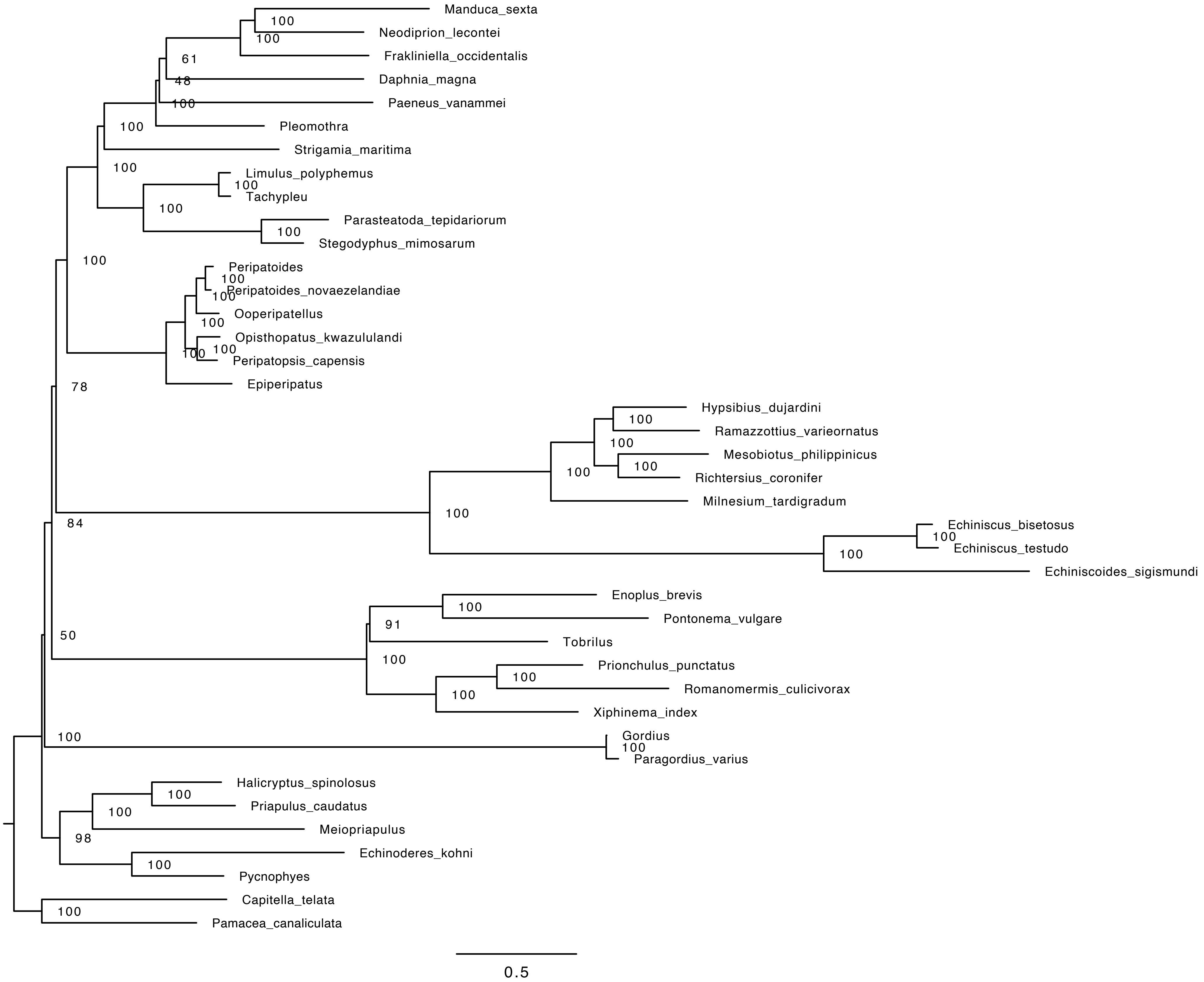

Manduca_sexta
100
Neodiprion_lecontei
100
Frakliniella_occidentalis
61
Daphnia_magna
48
Paeneus_vanammei
100
Pleomothra
100
Strigamia_maritima
100
Limulus_polyphemus
100
Tachypleu
100
Parasteatoda_tepidariorum
100
Stegodyphus_mimosarum
100
Peripatoides
100
Peripatoides_novaezelandiae
100
Ooperipatellus
100
Opisthopatus_kwazululandi
100
Peripatopsis_capensis
100
Epiperipatus
78
Hypsibius_dujardini
100
Ramazzottius_varieornatus
100
Mesobiotus_philippinicus
100
Richtersius_coronifer
100
Milnesium_tardigradum
100
84
Echiniscus_bisetosus
100
Echiniscus_testudo
100
Echiniscoides_sigismundi
Enoplus_brevis
100
Pontonema_vulgare
91
Tobrilus
50
100
Prionchulus_punctatus
100
Romanomermis_culicivorax
100
Xiphinema_index
100
Gordius
100
Paragordius_varius
Halicryptus_spinolosus
100
Priapulus_caudatus
100
Meiopriapulus
98
Echinoderes_kohni
100
Pycnophyes
Capitella_telata
100
Pamacea_canaliculata
0.5

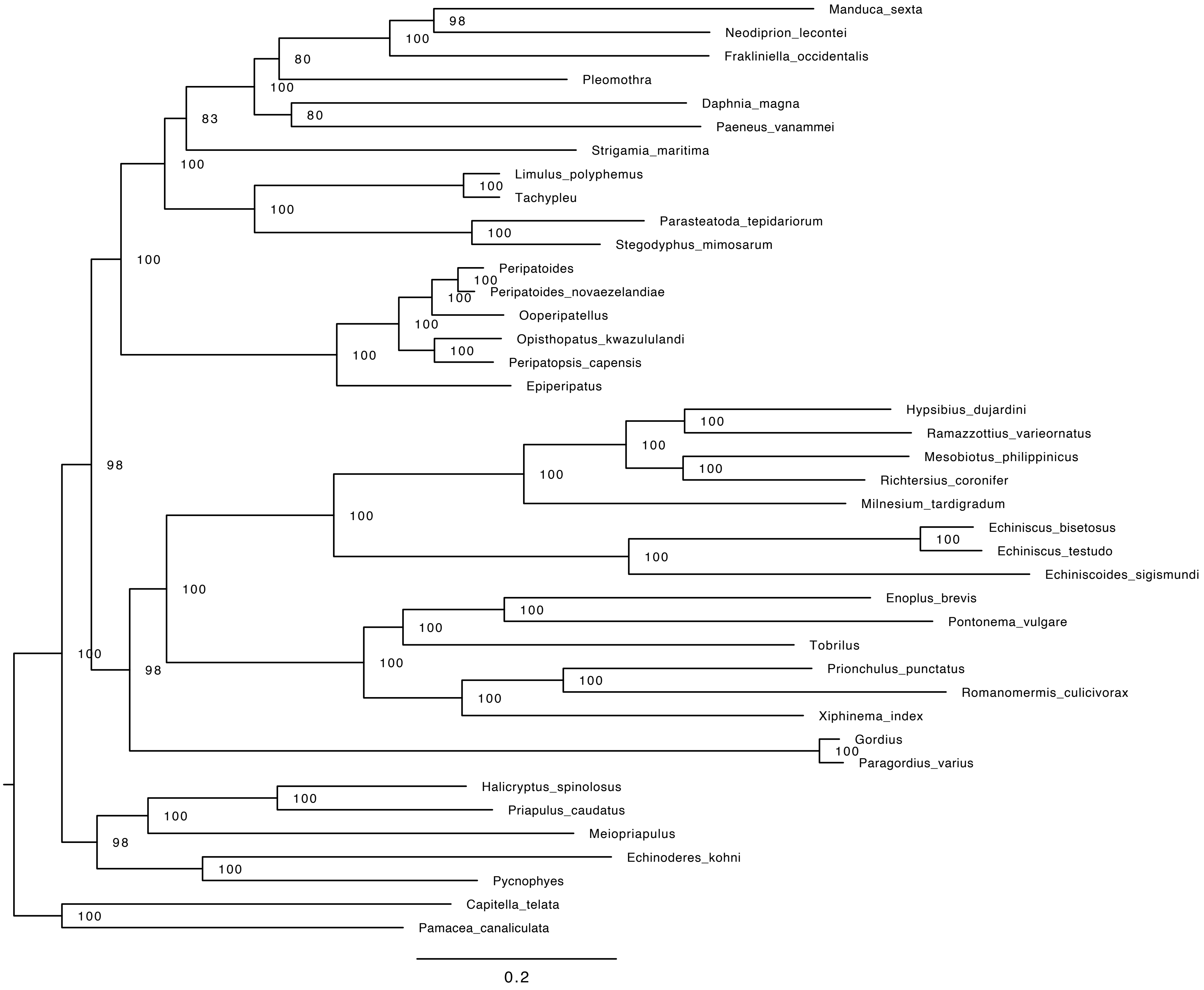
Manduca_sexta
98
Neodiprion_lecontei
100
Frakliniella_occidentalis
80
Pleomothra
100
Daphnia_magna
80
Paeneus_vanammei
83
Strigamia_maritima
100
Limulus_polyphemus
100
Tachypleu
100
Parasteatoda_tepidariorum
100
Stegodyphus_mimosarum
100
Peripatoides
100
Peripatoides_novaezelandiae
100
Ooperipatellus
100
Opisthopatus_kwazululandi
100
Peripatopsis_capensis
100
Epiperipatus
Hypsibius_dujardini
100
Ramazzottius_varieornatus
100
Mesobiotus_philippinicus
100
Richtersius_coronifer
100
Milnesium_tardigradum
98
100
Echiniscus_bisetosus
100
Echiniscus_testudo
100
Echiniscoides_sigismundi
100
Enoplus_brevis
100
Pontonema_vulgare
100
Tobrilus
100
Prionchulus_punctatus
100
100
Romanomermis_culicivorax
98
100
Xiphinema_index
Gordius
100
Paragordius_varius
Halicryptus_spinolosus
100
Priapulus_caudatus
100
Meiopriapulus
98
Echinoderes_kohni
100
Pycnophyes
Capitella_telata
100
Pamacea_canaliculata
0.2

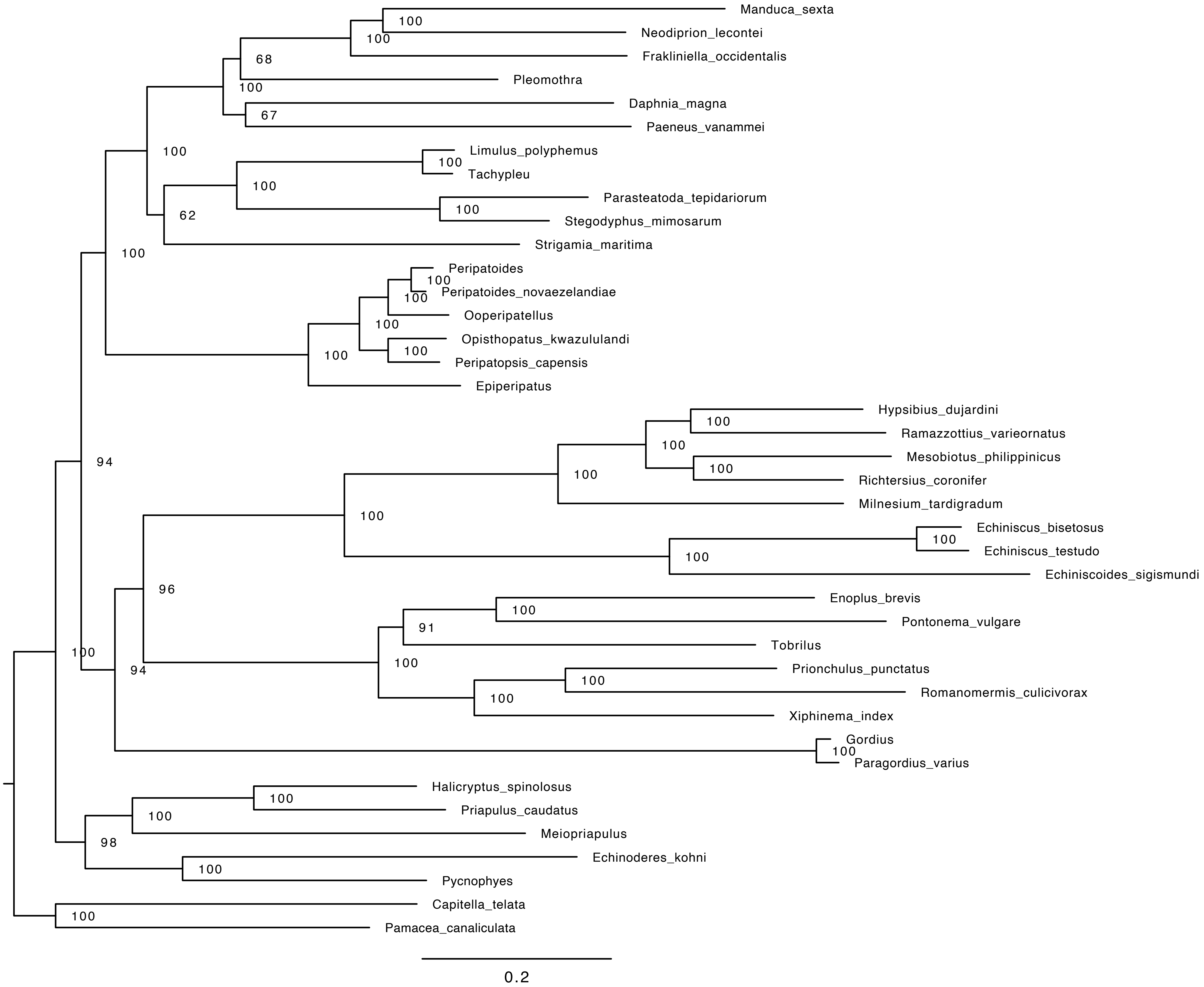
Manduca_sexta
Neodiprion_lecontei
Frakliniella_occidentalis
Pleomothra
Daphnia_magna
Paeneus_vanammei
Limulus_polyphemus
Tachypleu
Parasteatoda_tepidariorum
Stegodyphus_mimosarum
Strigamia_maritima
Peripatoides
Peripatoides_novaezelandiae
Ooperipatellus
Opisthopatus_kwazululandi
Peripatopsis_capensis
Epiperipatus
Hypsibius_dujardini
Ramazzottius_varieornatus
Mesobiotus_philippinicus
Richtersius_coronifer
Milnesium_tardigradum
Echiniscus_bisetosus
Echiniscus_testudo
Echiniscoides_sigismundi
Enoplus_brevis
Pontonema_vulgare
Tobrilus
Prionchulus_punctatus
Romanomermis_culicivorax
Xiphinema_index
Gordius
Paragordius_varius
Halicryptus_spinolosus
Priapulus_caudatus
Meiopriapulus
Echinoderes_kohni
Pycnophyes
Capitella_telata
Pamacea_canaliculata
100
68
67
62
94
96
91
98
0.2

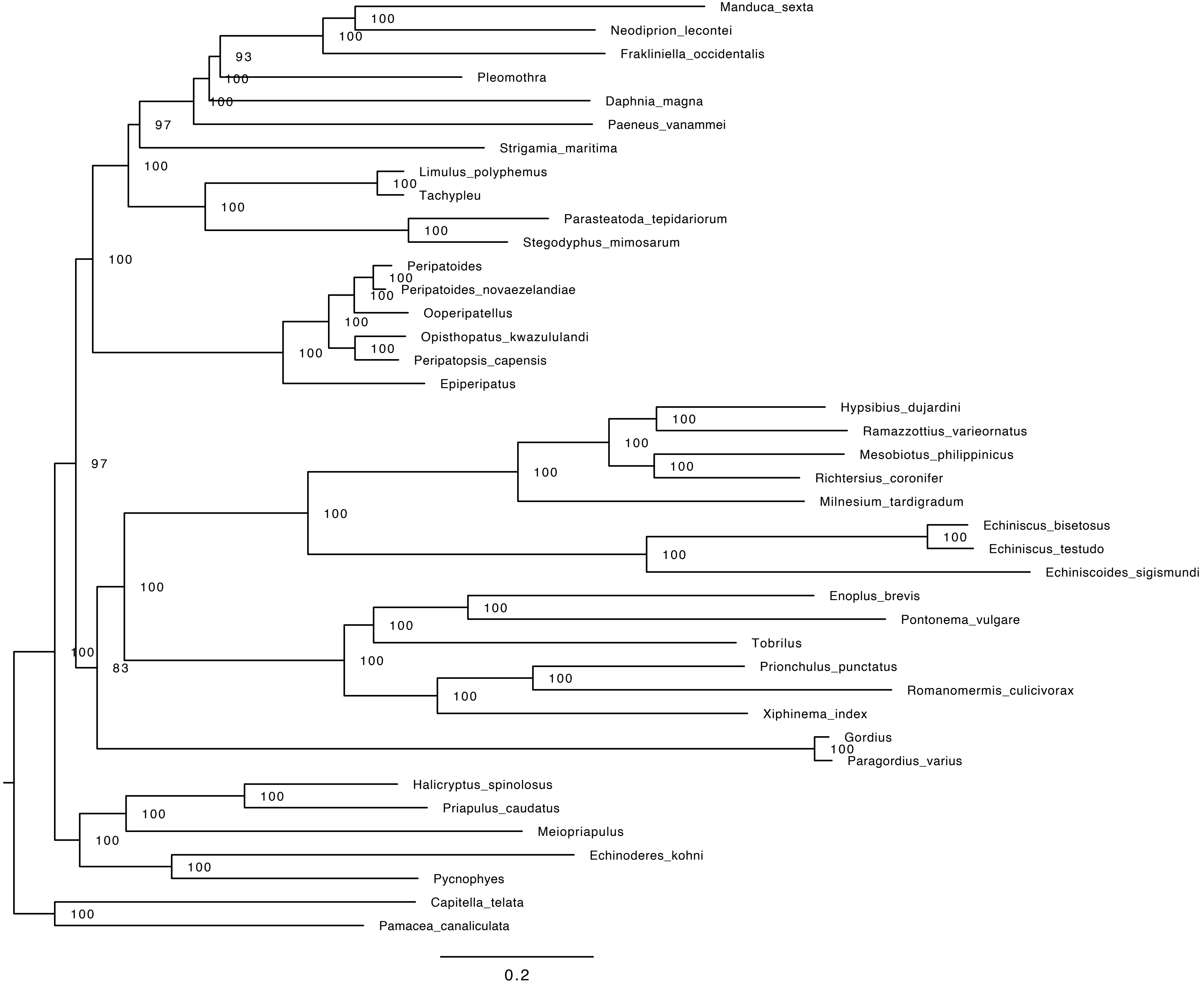

Manduca_sexta
Neodiprion_lecontei
Frakliniella_occidentalis
Pleomothra
Daphnia_magna
Paeneus_vanammei
Strigamia_maritima
Limulus_polyphemus
Tachypleu
Parasteatoda_tepidariorum
Stegodyphus_mimosarum
Peripatoides
Peripatoides_novaezelandiae
Ooperipatellus
Opisthopatus_kwazululandi
Peripatopsis_capensis
Epiperipatus
Hypsibius_dujardini
Ramazzottius_varieornatus
Mesobiotus_philippinicus
Richtersius_coronifer
Milnesium_tardigradum
Echiniscus_bisetosus
Echiniscus_testudo
Echiniscoides_sigismundi
Enoplus_brevis
Pontonema_vulgare
Tobrilus
Prionchulus_punctatus
Romanomermis_culicivorax
Xiphinema_index
Gordius
Paragordius_varius
Halicryptus_spinolosus
Priapulus_caudatus
Meiopriapulus
Echinoderes_kohni
Pycnophyes
Capitella_telata
Pamacea_canaliculata
100
93
97
83
0.2
